# A neural ensemble correlation code for sound category identification

**DOI:** 10.1371/journal.pbio.3000449

**Published:** 2019-10-01

**Authors:** Mina Sadeghi, Xiu Zhai, Ian H. Stevenson, Monty A. Escabí

**Affiliations:** 1 Department of Electrical and Computer Engineering, University of Connecticut, Storrs, Connecticut, United States of America; 2 Department of Biomedical Engineering, University of Connecticut, Storrs, Connecticut, United States of America; 3 Department of Psychological Sciences, University of Connecticut, Storrs, Connecticut, United States of America; Universidad de Salamanca, SPAIN

## Abstract

Humans and other animals effortlessly identify natural sounds and categorize them into behaviorally relevant categories. Yet, the acoustic features and neural transformations that enable sound recognition and the formation of perceptual categories are largely unknown. Here, using multichannel neural recordings in the auditory midbrain of unanesthetized female rabbits, we first demonstrate that neural ensemble activity in the auditory midbrain displays highly structured correlations that vary with distinct natural sound stimuli. These stimulus-driven correlations can be used to accurately identify individual sounds using single-response trials, even when the sounds do not differ in their spectral content. Combining neural recordings and an auditory model, we then show how correlations between frequency-organized auditory channels can contribute to discrimination of not just individual sounds but sound categories. For both the model and neural data, spectral and temporal correlations achieved similar categorization performance and appear to contribute equally. Moreover, both the neural and model classifiers achieve their best task performance when they accumulate evidence over a time frame of approximately 1–2 seconds, mirroring human perceptual trends. These results together suggest that time-frequency correlations in sounds may be reflected in the correlations between auditory midbrain ensembles and that these correlations may play an important role in the identification and categorization of natural sounds.

## Abstract 2

Animals and humans can readily categorize sounds into behaviorally relevant categories, but the neural mechanisms responsible for category formation are unknown. Neural recordings and modelling reveal that coordinated firing in auditory midbrain circuits provides a robust neural signal for decoding the identity of natural sounds and categories.

## Introduction

In the auditory periphery, neurons encode sounds by decomposing stimuli into cardinal physical cues such as sound pressure and frequency, which retain detailed information about the incoming sound waveform. In midlevel auditory structures, such as the inferior colliculus (IC), sounds are further decomposed into higher-order acoustic features such as temporal and spectral sound modulations. Rather than firing when a frequency is simply present in the sound, IC neurons also respond selectively to spectro-temporal structure in the envelopes of frequency channels [1]. In natural sounds, these spectro-temporal modulations are highly structured and varied, and the envelopes are correlated both across frequencies and time [2–5]. While low-level cues such as the sound spectrum contribute to many auditory tasks, including sound localization and pitch perception [6,7], spectral cues alone are insufficient for identifying most environmental sounds. Manipulating higher-order statistics related to the spectro-temporal modulations of sounds can dramatically influence recognition [2]. Temporal modulations contribute to the familiarity and recognition of natural sounds such as running water sounds [8,9], and temporal coherence across frequencies plays a central role in auditory stream segregation [10]. Here, we examine to what extent correlations in neural ensembles vary with natural sounds and to what extent both neural and sound correlations may be useful for sound recognition. Although correlations are often thought to lead to less efficient representations of individual sensory stimuli [11,12], here, we find evidence that correlation statistics may contribute to sound recognition and the formation of acoustic categories.

The spectro-temporal correlation structure of amplitude modulations in sounds is known to contribute to auditory perception [2] and strongly modulates single-neuron activity in the auditory midbrain [1]. For single neurons, correlated sound structure can improve signal detection [13] and coding of spectro-temporal cues [14] and also activates gain control mechanisms [15,16]. However, it is currently unclear how sound correlations shape the response correlations of neural ensembles. Pairs of neurons at multiple levels of the auditory pathway show correlated firing that strongly depends on the spatial proximity of neurons, receptive field similarity, and behavioral state [17–19], but it remains to be seen whether these correlations are stimulus-dependent and whether they are detrimental or beneficial for coding. Theories based on efficient coding principles have proposed that stimulus-driven correlations should be minimized in order to minimize redundancies in the neural representation [11,12], and noise correlations, which reflect coordinated firing in an ensemble of neurons that is not directly related to the sensory stimulus, are thought to directly limit the encoding of sensory information [20,21]. On the other hand, correlations between neurons may be functionally important and have previously been considered as plausible mechanisms for sound localization [22] and pitch identification [23,24]. Here, we consider a more general role for stimulus-driven ensemble correlations and test whether they may be useful for sound recognition in general.

The IC receives convergent input from various brainstem nuclei and has the potential to consolidate ascending auditory information, which is ultimately relayed to the thalamus and cortex. Furthermore, the IC is selective for various structural features in natural sounds and has been proposed to form a compact and efficient midlevel auditory representation [3,19,25–27]. As such, it is believed to be necessary for extracting spectro-temporal cues that underlie sound recognition. Here, we test the hypothesis that sound-correlation statistics alter the correlated firing between frequency-organized neuron ensembles in the IC and that these stimulus-driven correlations can be decoded to identify individual sounds. Using an auditory model and accompanying neural recordings, we then test whether sound-correlation statistics can be used to identify sound categories. Collectively, the data suggest stimulus-driven correlations between neuron ensembles in the auditory midbrain may provide a signature for downstream neurons to recognize and categorize natural sounds.

## Results

### Decoding neural ensemble correlation statistics to recognize sounds

We used multichannel, multiunit neural recordings in the auditory midbrain (IC) of unanesthetized rabbits to characterize and determine whether neural correlations between recording sites in the IC are affected by the correlation structure of natural sounds and whether such neural ensemble statistics could potentially contribute to sound recognition.

**Fig 1:**
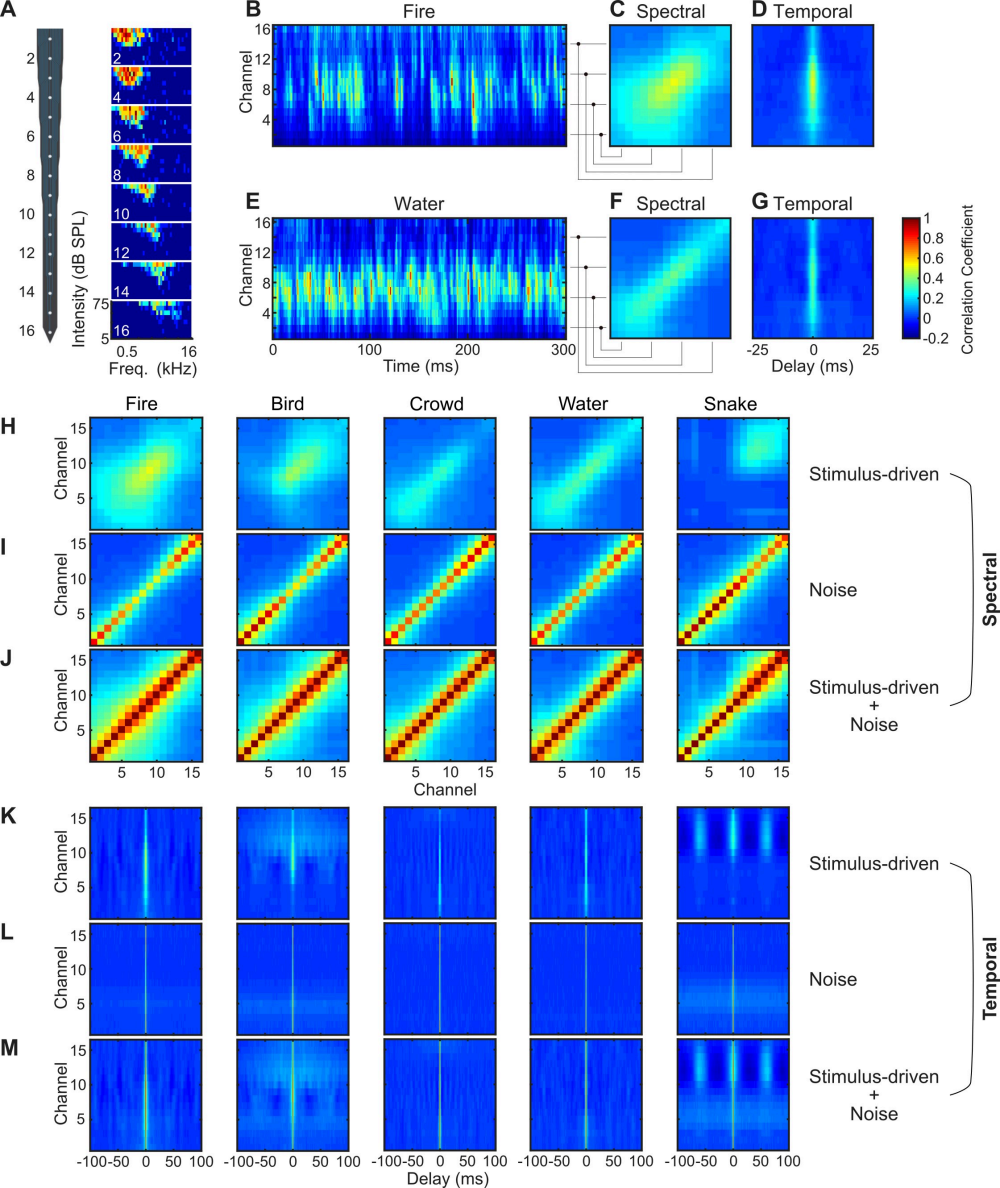
Neural ensemble correlation statistics for an auditory midbrain penetration site. (A) Neural recording probe and the corresponding frequency response areas at 8 staggered recording sites show tonotopic organization (red indicates high activity; blue indicates low activity). aMUA for the 16 recording channels for a (B) fire and (E) water sound segment (red indicates strong response; blue indicates weak response). The spectral (C = fire; F = water) and temporal (D = fire; G = water) neural ensemble correlation for the penetration site. Stimulus-driven spectral (H) and temporal (K) correlations of the recording ensemble show distinct differences and unique patterns across the five sounds tested. Spectral (I) and temporal (L) noise correlations recorded during the same sound delivery sessions are substantially less structured (diagonalized spectrally and restricted across time) and show little stimulus dependence. The total spectral and temporal ensemble correlations (stimulus-dependent + noise) are shown for the same site and sounds in J and M, respectively. Additional examples penetration sites are provided in S2–S4 Figs. Figure data and related code are available from http://dx.doi.org/10.6080/K03X84V3. aMUA, analog multiunit activity; SPL, sound pressure level; Freq., frequency.

The spatiotemporal correlation statistics from an example penetration site demonstrate the diversity of neural ensemble correlation statistics observed in a frequency-organized recording site. As expected for the principal nucleus of the IC, frequency responses areas are tonotopically organized, varying from low to high frequency with recording depth (Fig 1A; approximately 0.5–8 kHz; low frequencies more dorsal, high frequencies more ventral), and, for this reason, spatial correlations are referred to as spectral correlations in what follows. Response neurograms (averaged across trials) are shown for a fire sound (Fig 1B) and a water sound (Fig 1E) for this recording site, and the stimulus-driven ensemble correlations are estimated by correlating the outputs of each recording channel across independent response trials (Fig 1C, 1D, 1F and 1G; Materials and Methods). In general, neural correlation statistics to natural sound are highly diverse across sounds. The stimulus-driven spectral correlation matrix (at zero time lag) reflects the instantaneous correlation of the neural activity between different frequency-organized recording sites (Fig 1C for fire; 1F for water; cochleograms and corresponding neural activity patterns are shown for this site in S1 Fig). In contrast, the trial-shuffled autocorrelograms for each recording site reflect the temporal correlation structure of the neural activity at different recording locations (Fig 1D for fire; 1G for water).

The stimulus-driven neural correlations of the same penetration site are shown for natural sound recordings of a crackling fire, a bird chorus, crowd noise, running water, and a rattling snake, all of which contain a mixture of the target sounds and natural ambient noises (available for listening, S1–S5 Sounds). Across sounds, spatial/spectral (Fig 1H) and temporal (Fig 1K) correlations are quite diverse and reflect stimulus-dependent structure. For instance, in this example penetration site, the water and crowd sounds have similar stimulus-driven spectral correlation matrices, with the highest correlations localized to neighboring low-frequency channels (<4 kHz, along the diagonal, slightly higher range for water). Correlations are much more extensive and widespread for the fire sound. Nearby channels are still highly correlated (along the diagonal), but distant recording channels can also have high correlations. The bird chorus and rattling snake sound, by comparison, have their own distinct correlation patterns, with the strongest correlations occurring between sites with best frequencies above approximately 1 and 2 kHz, respectively. In general, stimulus-driven spectral correlations exhibit strong stimulus-dependent structure and high diversity across recording locations (additional examples, S2–S4 Figs)

The stimulus-driven temporal correlations also have unique stimulus-dependent pattern and unique timescales for each sound (Figs 1K and S2–S4). The temporal correlations for the crowd, water, and fire sounds are relatively fast (3.0 ms, 3.0 ms, and 3.9 ms width at 50% of the maximum; 5.9 ms, 6.9 ms, and 11.8 ms width at 10% of the maximum). The bird chorus has a similar sharp temporal correlation (5.9 ms half width), but it also contains a broader and substantially slower component (161.2 ms, 10% width). By comparison, although the rattling snake sound also has a relatively precise temporal correlation at zero lag (7.9 ms, 50% width; 20.6 ms, 10% width), the neural ensemble generates a periodic pattern for high-frequency sites with a period of approximately 50 ms, reflecting the structure of the rattling at approximately 20 Hz. Furthermore, these neural correlations were stimulus-driven and frequency-dependent since they were partly predicted by the spectral correlations from a peripheral auditory model (see Materials and Methods; average *r* = 0.3 ± 0.03, *t* test, *p* < 0.01; S5 Fig).

We next decomposed the neural correlations by measuring the noise-driven correlations of the neural ensemble (see Materials and Methods). This allows for determining how noise and stimulus-driven correlations individually contribute to sound recognition. In contrast to stimulus-driven correlations, which are highly structured in both time and frequency, noise correlations mostly lack stimulus-dependent structure. For the spectral domain, noise correlations are largely diagonalized and limited largely to neighboring channels (Figs 1I and S2–S4). Temporal noise correlations (Figs 1L and S2–S4) are fast, lasting only a few milliseconds and localized about zero millisecond delay. Noise correlations were also generally stronger than the corresponding signal correlations (spectral Signal-to-Noise Ratio [SNR]: range = −26.0 to −0.9 dB, −11.0 ± 7.2 mean ± SD; temporal SNR: range = −26.1 to −4.1 dB, −6.0 ± 8.2 mean ± SD), indicating that much of the structure in correlations between channels is not stimulus-dependent. The total response correlation (Fig 1J for spectral; Fig 1M for temporal) had a substantial stimulus-independent component, particularly for the spectral correlations, which tended to have a lower SNR.

**Fig 2:**
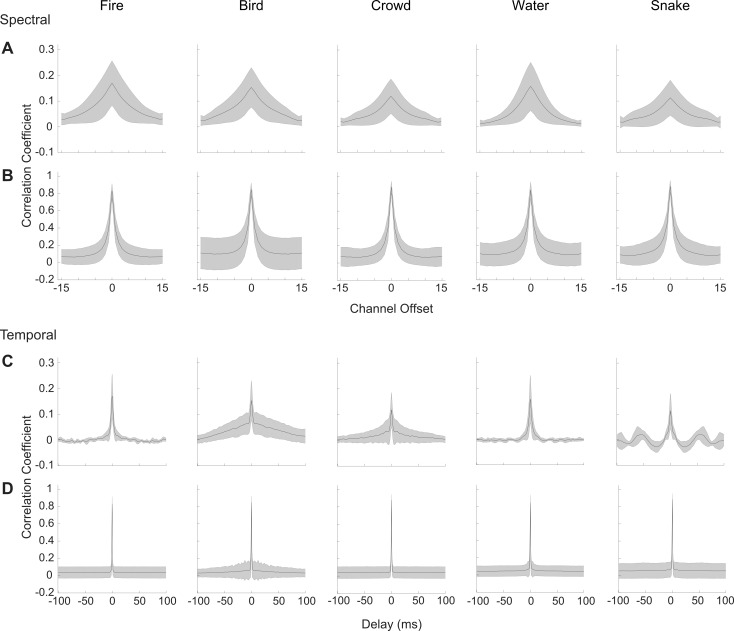
Average stimulus-driven and noise-driven neural correlations. Summary results showing the average stimulus-driven (A = spectral, C = temporal) and noise-driven (B = spectral; D = temporal) neural correlations across *N* = 13 penetration sites (*N* = 4 and 9, from two animals). To allow for averaging across recording sites with different best frequencies, the spectral and temporal correlation matrices (as for A, C, and Fig 1H and 1K) are collapsed across their principal dimension (channel offset for spectral and time lag for temporal) prior to averaging. The average noise-driven correlations are more compact, being restricted in both time and frequency, and have less structure across sounds than the corresponding stimulus-driven correlations. Figure data and related code are available from http://dx.doi.org/10.6080/K03X84V3.

As for the example, stimulus-driven correlations across our recordings exhibit stimulus-dependent structure, whereas noise correlations show little stimulus dependence and are limited primarily to neighboring channels spectrally and brief epochs in time. To measure the average noise and stimulus correlations across penetration sites, which have mismatched frequency range, we collapsed the stimulus and noise correlations along the frequency dimension in order to obtain a frequency-independent measure of the correlated activity. Population average results are shown in Fig 2. The spectral noise correlations (collapsed along the diagonal) are consistent across sounds and more restricted in frequency (Fig 2B) than the corresponding stimulus-driven spectral correlations (Fig 2A). This indicates that noise correlations are not broadly diffused across recording locations and are largely independent of the stimulus. Stimulus-driven correlations, by comparison, are broader and can be observed between more distant recording locations. Similarly, temporal noise correlations exhibit a brief peak around zero lag and lack stimulus-dependent structure (Fig 2D). The stimulus-driven temporal correlations (Fig 2C), by comparison, also have a brief peak at zero lag; however, they also exhibit a broader and slower correlation component and are substantially more dependent on the stimulus. For instance, a relatively broad and slow component is observed for the bird vocalization, and a periodic component at approximately 20 Hz is observed for the rattling snake sound.

**Fig 3:**
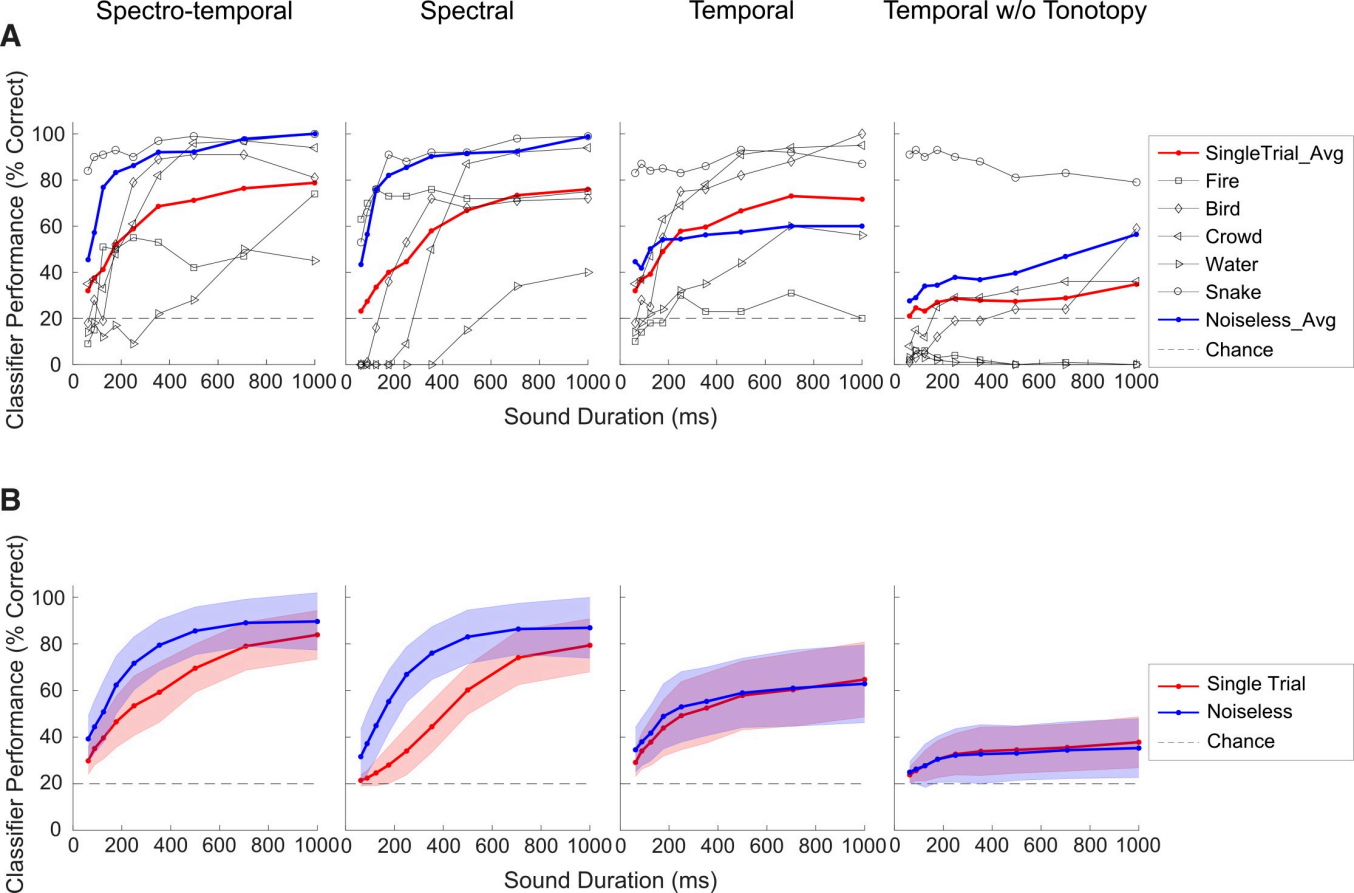
Using neural ensemble correlation statistics to identify sounds. (A) Single-trial classification results for the penetration site shown in Fig 1. The average single-trial classifier performance (red curve) and the performance for each individual sound (black lines) are shown as a function of the sound duration for four classifiers. Blue curves designate upper bound on performance based on a noiseless classifier (see Materials and Methods). In all cases, classifier performance improves with sound duration. The combined spectro-temporal classifier has the highest performance, followed by the spectral and temporal classifiers. Removing the tonotopic ordering of recording sites for the temporal classifier for this recording location (far right) substantially reduces its performance. (B) Average performance across *N* = 13 IC penetration sites (*N* = 4 and 9, from two animals) for each of the four classifiers shown as a function of sound duration for the single-trial (red) and noiseless (blue) classifiers. Red and blue bands represent SD. The average performance for each individual sound and classifier is provided in S6 Fig. Figure data and related code are available from http://dx.doi.org/10.6080/K03X84V3. IC, inferior colliculus; w/o, without.

Since the structure of the stimulus-driven correlations are highly diverse, we next quantify the extent to which these response statistics could be used to identify sounds (Fig 3). We use a single-trial classifier based on a Bayesian model of the correlations to determine whether the spectral or temporal neural correlation structure could distinguish among the five sounds delivered (see Materials and Methods). Neural classifier results are shown for the penetration site shown in Fig 1 (Fig 3A, red curve). Upper bounds on the classifier performance are also approximated with a noiseless classifier (Fig 3A, blue curves) that averages the validation data across trials, thus removing much of the trial-to-trial variability (i.e., “noise” correlations) and isolating stimulus-driven structure (see Materials and Methods, S1 Text for derivation). For short durations (62.5 ms), the spectro-temporal classifier performance for each sound is quite variable, although the average performance is above chance level (20%). As expected, the individual sound (black curves) and average (red curve) classifier performance improves with the duration of the recording. In isolation, the spectral- and temporal-only single-trial classifiers have similar, although slightly lower, performances. Furthermore, for both spectral and spectro-temporal classifiers, the noiseless classifier performance (blue curve) is higher than the single-trial classifier (red curve). By comparison, for temporal classifier, the single-trial and noiseless classifiers have similar performances, indicating that the temporal classifier performance is not strongly affected by noise correlations. This is consistent with the observation that noise correlations, which are present in single-trial activity, are largely stimulus-independent and can limit the classification accuracy. As such, the noiseless classifier approximates an upper bound on the performance for this particular classifier when noise is not present.

It is possible that tonotopic ordering of the temporal correlation matrix provides information that contributes to the temporal classifier performance. The snake and bird chorus sounds, e.g., both have stronger temporal correlations at high frequencies (Fig 1K), which could provide valuable frequency-dependent information for classification. Indeed, altering tonotopy by manipulating the frequency ordering of sounds has been shown to impair pitch perception and vowel recognition [7,28]. To measure the contribution of purely temporal correlations, we distorted the tonotopic ordering from the temporal correlation matrix by randomizing the channel ordering. Doing so substantially reduces the temporal classifier performance from 72% to 35% for the longest sound duration measured (1 s; Fig 3A). Thus, removing the tonotopic ordering to isolate purely temporal correlations leads to a reduction in the classifier accuracy.

Across all penetration sites, we find that, just as with the single example, neural correlation structure is highly informative and can be used to recognize sounds. Across all sounds and classifiers, the average performance is above chance (red curves in Fig 3B; *t* test, *p* < 0.01, tested at 1 s duration) and improves with increasing sound duration (Fig 3B). Furthermore, as for the example recording site, performance is degraded substantially when tonotopic ordering is removed (Fig 3B, far right). Similar performance improvements are observed when sounds are analyzed individually (S6 Fig), and, although the spectral and temporal classifier performances are similar on average (Fig 3), they can be very different for different sounds (S6 Fig). These differences suggest that spectral and temporal correlation statistics can contribute differentially and independently to sound identification and that, when combined, they can improve classification performance

### Differences in correlations statistics are not due to sound spectrum alone

One possible explanation for the stimulus-dependent neural correlations is that these statistics may be driven by the spectral content of a sound. For instance, channels tuned to frequencies with high power could potentially have stronger correlations. Since sound spectra can vary extensively as a function of direction (head and pinnae filtering) and acoustical absorption properties of the environment, the correlations would thus be influenced by factors not related to the sound identity. On the other hand, if stimulus-driven correlations reflect other sound cues, such as fine structure and temporal modulation cues that neurons throughout the auditory pathway respond to [29], these statistics may allow for a more stable neural representation.

To examine the extent to which the spectrum may be driving the observed correlations, we repeated the sound identification task using sound variants that were equalized to have a matched power spectrum (see Materials and Methods; S7 Fig). This manipulation assures that spectrum cues are the same across sounds and that only fine structure and temporal modulation cues differ. Perceptually, these sounds are readily identified as the original (available for listening; S6–S10 Sounds) despite the fact that their spectra are identical and that the original and equalized spectra can deviate by as much as 60 dB (S7 Fig).

**Fig 4:**
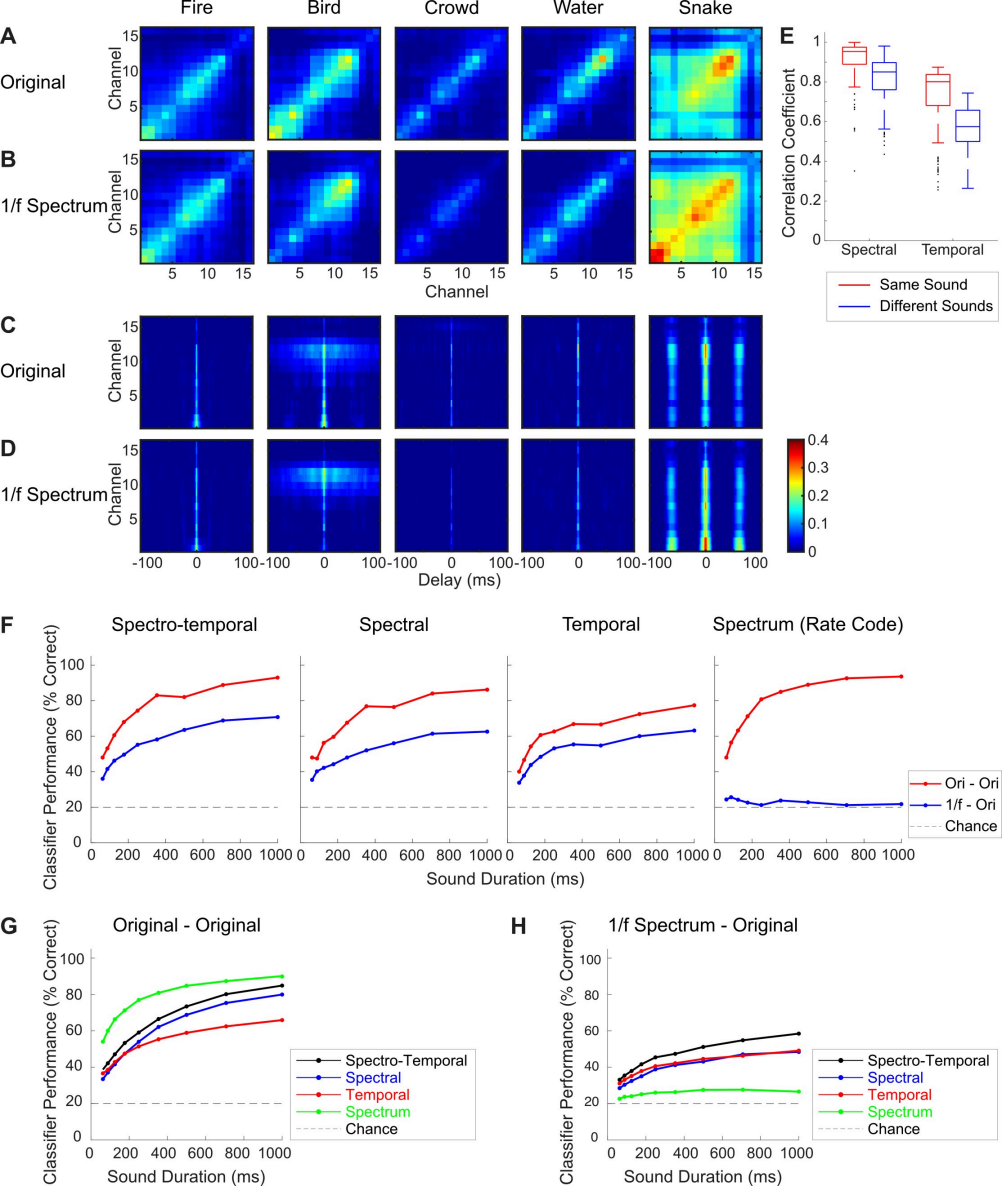
Neural ensemble correlation statistics for sound variants with equalized power spectrum. The spectral (A and B) and temporal (C and D) correlations of a single IC recording site show similar structure between the original (A and C) and 1/f equalized (B and D) sounds (Pearson correlation coefficient, *r* = 0.92 for spectral; *r* = 0.72 for temporal; averaged across five sounds). (E) Across penetration sites (*N* = 11 total; *N* = 3 and 8 from two animals), the neural correlations of the original and spectrum-equalized variants have an average Pearson correlation coefficient of *r* = 0.95 ± 0.02 for spectral and *r* = 0.80 ± 0.02 for temporal when the comparison is between same sounds (e.g., original fire versus 1/f fire sounds; red). Across-sound comparisons (e.g., original fire versus 1/f water sound; blue) show a reduced correlation (*r* = 0.85 ± 0.02 for spectral; *r* = 0.57 ± 0.01 for temporal). (F) Single-trial classification results (averaged across five sounds) for the above penetration site obtained for the original and spectrum-equalized sounds. The model is trained using the responses to the original sounds, while the validation data are from the responses to the original (red) or the spectrum-equalized (blue) sounds (see Materials and Methods). The spectrum-equalized (1/f) condition shows slightly lower performance for spectro-temporal, spectral, and temporal classifiers, while the spectrum (rate code) classifier is near chance. Average performance versus sound duration across *N* = 11 IC penetration sites for each of the four classifiers. Classification performance is shown for the original (G) and spectrum-equalized (H) sound responses. Figure data and related code are available from http://dx.doi.org/10.6080/K03X84V3. IC, inferior colliculus; Ori, original.

Fig 4 shows that the neural correlations for both the original sounds and spectrum-equalized conditions of a single recording location are remarkably similar (Pearson correlation coefficient, *r* = 0.92 ± 0.01 for spectral, *r* = 0.72 ± 0.07 for temporal; mean ± SE). Across recording locations, the neural correlations for the original and equalized sounds have an average Pearson correlation coefficient of 0.95 ± 0.02 for spectral and 0.80 ± 0.02 for temporal (mean ± SE). Overall, this suggests that the much of the neural correlation structure is driven by fine structure and modulation cues and is not solely determined by the sound spectrum.

Next, we tested whether the neural correlations are sufficiently preserved despite the spectrum equalization to allow neural decoding. For this paradigm, the classifier was trained using responses to the original sounds (as for Fig 1), whereas the validation data were obtained from either the spectrum-equalized or the original sound response (as a control). For the site of Fig 4A–4D, the accuracy of the spectral, temporal, and spectro-temporal classifiers is slightly reduced for the equalized sounds (Fig 4F; blue when compared to the original sound, red), and the maximum performance exceeds chance (*p* < 0.05, *t* test) and remains high (63% for spectral, 63% for temporal, 71% for spectro-temporal). Furthermore, although the spectrum (rate) classifier achieves very high accuracy approaching 94% for the original sound, accuracy drops to chance level when the spectrum is equalized (Fig 4F, far right).

Similar results are observed for the entire set of recordings (Fig 4G and 4H). For the three correlation-based classifiers (spectral, temporal, and spectro-temporal), residual information remains when the spectrum is equalized. All three classifiers perform above chance for 1 s duration when the spectrum is equalized (Fig 4H; spectral = 48%, temporal = 49%, spectro-temporal = 58%; *p* < 0.01, *t* test), and classification accuracy improves with increasing duration. In sharp contrast, although average accuracy of the spectrum classifier exceeds all three correlation classifiers for the original sound (Fig 4G, green curve; 90.0 ± 2.0%, mean ± SE), accuracy drops to near chance (Fig 4H, green curve; 27.0 ± 1.0%, mean ± SE) and does not substantially improve with sound duration when the spectrum cues are removed.

### Correlation statistics of natural and man-made sound categories

After establishing that neuron ensembles in IC have highly structured spatiotemporal correlation statistics, we now aim to determine to what extent these correlations are inherited from the correlations in the sounds themselves. We use a dynamic auditory model to characterize the structure of the time-averaged and time-varying spectro-temporal correlations in an assortment of 13 natural sound categories and then determine their potential contribution towards sound category identification.

#### Average correlation statistics and diversity

**Fig 5:**
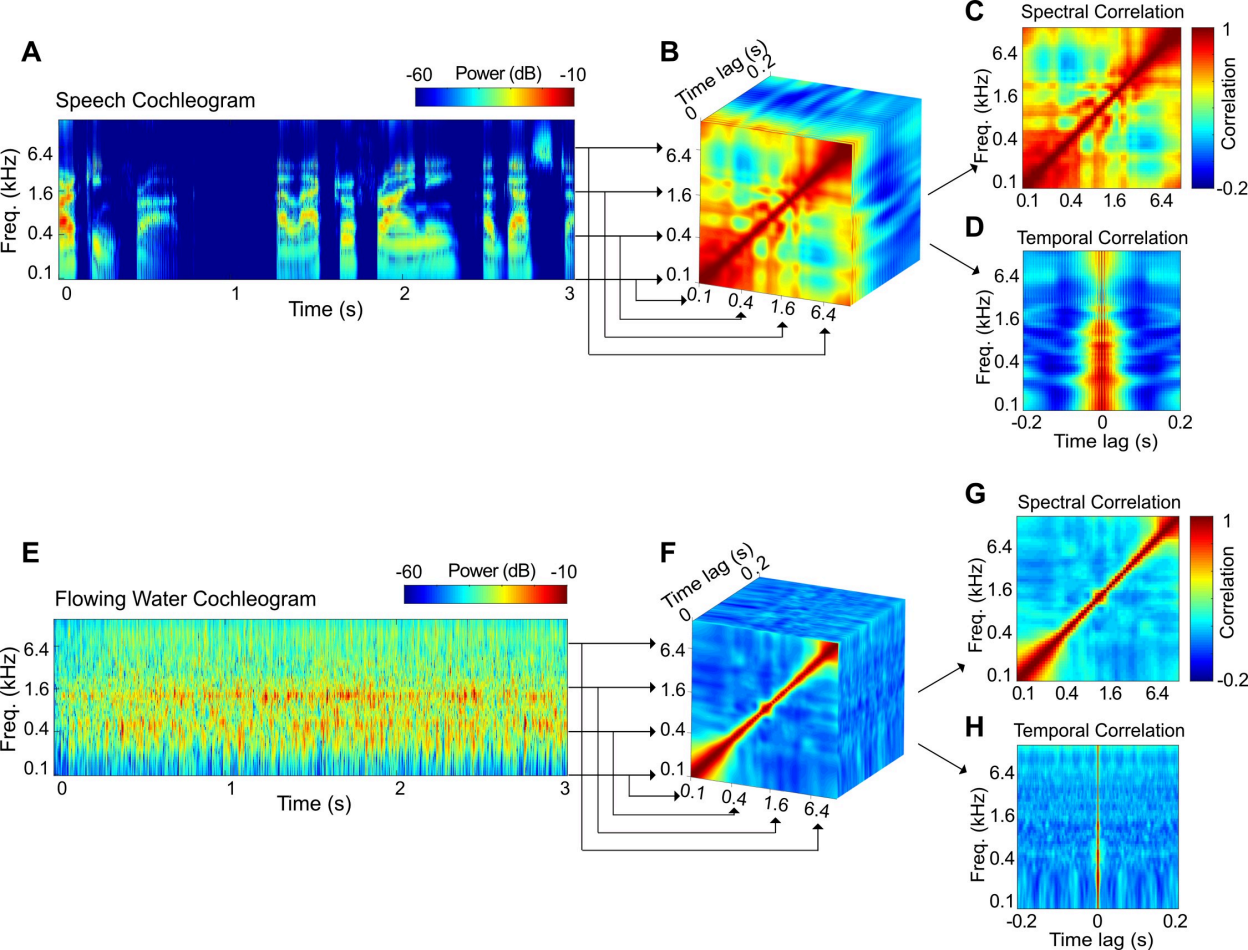
Measuring the average correlation structure of natural sounds. The procedure is illustrated for a speech and a flowing water sound. The spectro-temporal correlations are obtained by cross-correlating the frequency-organized outputs of a cochlear model representation (A, E). The resulting spectro-temporal correlation matrices (B, F) characterize the correlations between frequency channels at different time lags. The spectro-temporal correlations are then decomposed into purely spectral (C, G) or temporal (D, H) correlations. Speech is substantially more correlated across frequency channels, and its temporal correlation structure is substantially slower than for the water sound. Figure data and related code are available from http://dx.doi.org/10.6080/K03X84V3. Freq., frequency.

We first evaluated the correlations between modulations of different frequency-selective outputs of a cochlear model (Fig 5; see Materials and Methods). The time-averaged spectro-temporal correlations (Fig 5B and 5F) highlight distinct acoustic differences between sounds. At zero lag, the spectro-temporal correlations reflect the instantaneous or spectral correlations between different frequency channels (Fig 5C and 5G), while correlations within the same frequency channels at different time lags reflect the temporal correlation structure of each sound (Fig 5D and 5H). For example, the spectral correlation structure of speech is relatively broad, reflecting the strong comodulation between frequency channels. This contrasts the running water excerpt, in which the correlations are largely diagonalized, with minimal correlation between distant frequency channels. The temporal correlation structure in speech exhibits a relatively slow temporal structure (64 ms half width), which reflects the relatively slow time-varying structure of speech elements and words [30,31]. By comparison, the water sound has a relatively fast temporal correlation structure (4 ms half width) that is indicative of substantially faster temporal fluctuations in the sound power.

**Fig 6:**
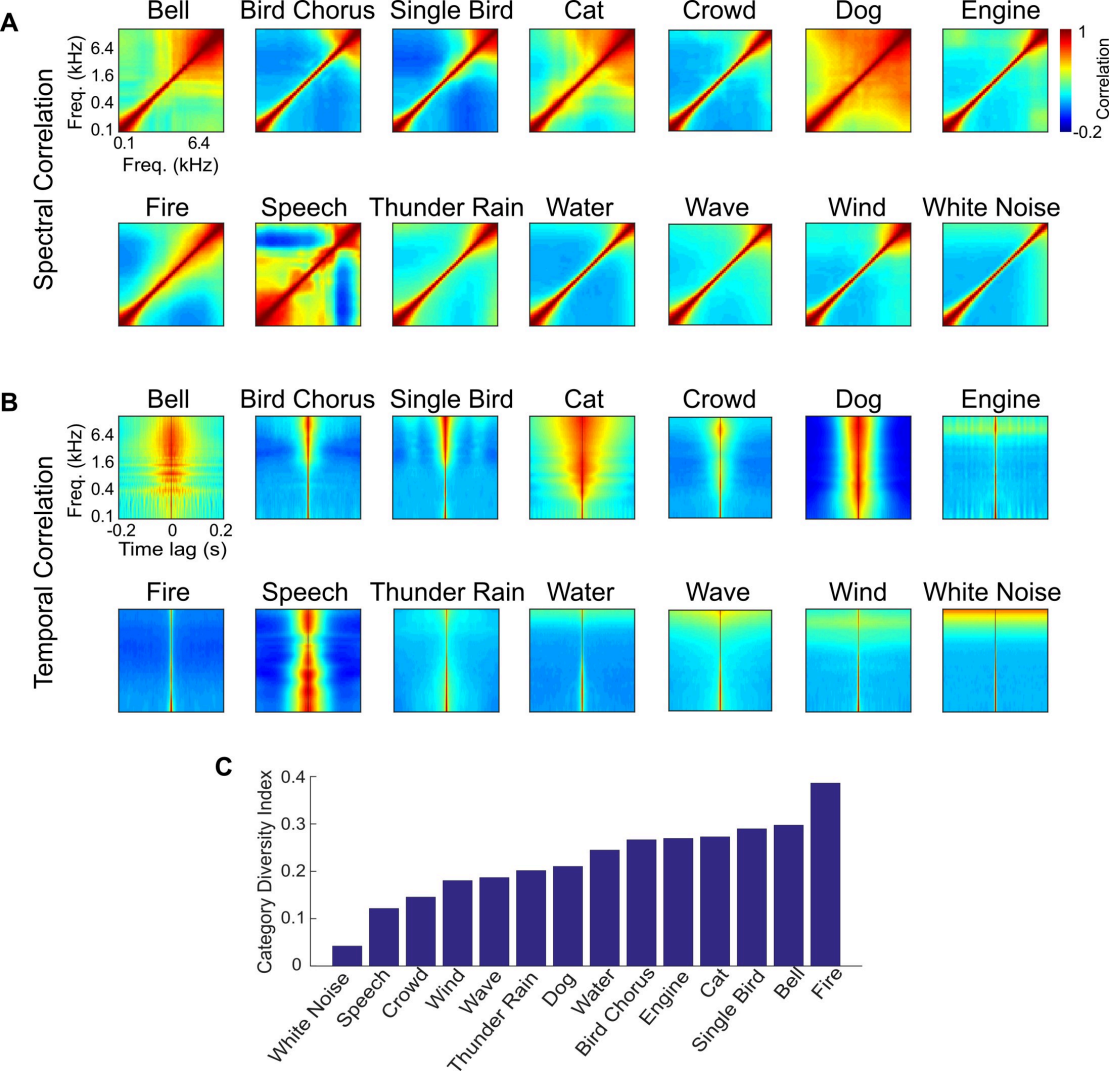
Sound-correlation statistics for the 13 sound categories and white noise. The category average (A) spectral correlation matrix and (B) temporal correlations show unique differences among the 13 sounds examined. (C) The CDI quantifies the variability of the correlation statistics for each category. A CDI of 1 indicates that the sound category is diverse (the correlation statistics are highly variable between sounds), while 0 indicates that the category is homogenous (all sounds have identical correlation statistics). A detailed list of sounds and sources used is provided in S1 Table. Figure data and related code are available from http://dx.doi.org/10.6080/K03X84V3. CDI, category diversity index; Freq., frequency.

The average spectral (Fig 6A) and temporal (Fig 6B) correlations of 13 sound categories and white noise (as a control) reflect conserved acoustic structure in each of the categories, and, for the categories tested here, this structure is highly diverse. Certain sound categories, particularly background sounds such as those from water (rain, running water, waves) and wind, have relatively restricted spectral correlations (diagonalized) and relatively fast temporal correlation structure (impulsive; correlation half width: water = 3.9 ms; wind = 3.6 ms). Such a diagonalized and fast temporal structure reflects the fact that these sounds are relatively independent across frequency channels and time. Note that because of the bandwidth of the overlapping filters in the cochleogram, white noise has similar, restricted spectral correlations rather than perfectly uncorrelated frequency channels. Other sounds such as isolated vocalizations (e.g., cat, dogs, and speech) have more varied and extensive spectral correlation that is indicative of strong coherent fluctuations between frequency channels. Such sounds also have relatively slow temporal correlation structure (correlation half width: speech = 64.9 ms; dogs = 76.3 ms; cats = 117.3 ms), indicating slow dynamics associated with the production of vocalizations.

Although the average statistics illustrate differences between categories that could facilitate recognition, each individual sound in a category can be statistically quite different from the average, which could limit the usefulness of such statistics for recognition. Sound categories in which the correlation statistics exhibit little diversity from sound to sound may be easier to identify, while sounds with large amount of diversity might be more difficult to identify. We thus developed a category diversity index (CDI) to quantify the diversity in the correlation structure within each of the sound categories (Fig 6C). Of all sounds in our database, white noise has the smallest diversity, which is expected given that white noise is wide-sense stationary. Of the natural sounds, speech has the lowest diversity (CDI = 0.12). Although this is unexpected, it likely reflects the fact that the sound segments used in our database consisted of different speech excerpts from a single male speaker. More generally, there is no clear distinction or trend between different classes of sounds. For instance, the diversity indices for isolated vocalization categories are quite varied. Bird songs have a relatively high CDI of 0.29, and barking dogs have intermediate values (0.21). Similarly, the diversity of background sounds is quite varied, ranging from highly diverse categories such as fire (CDI = 0.39) to less diverse categories such as crowd noise (CDI = 0.15) and wind (CDI = 0.18). Although such trends partly reflect biases in the selection of sounds in the database, they also likely reflect acoustic properties that are unique to each sound category.

#### Short-term correlation statistics and stationarity

While the average correlation statistics provide some insights of the structural differences between sound categories, many sounds, such as vocalizations, exhibit nonstationary structure with complex temporal dynamics and are not well-described by time-averaged correlation statistics. Here, we use time-varying, modified short-term correlations [32] to characterize nonstationary structure. Computations involving time-localized and continuously varying correlations may be more plausible than time-averaged correlations because lemniscal auditory neurons integrate sounds over restricted integration time windows (approximately 10 ms in the midbrain to 100 ms in cortex [19]).

**Fig 7:**
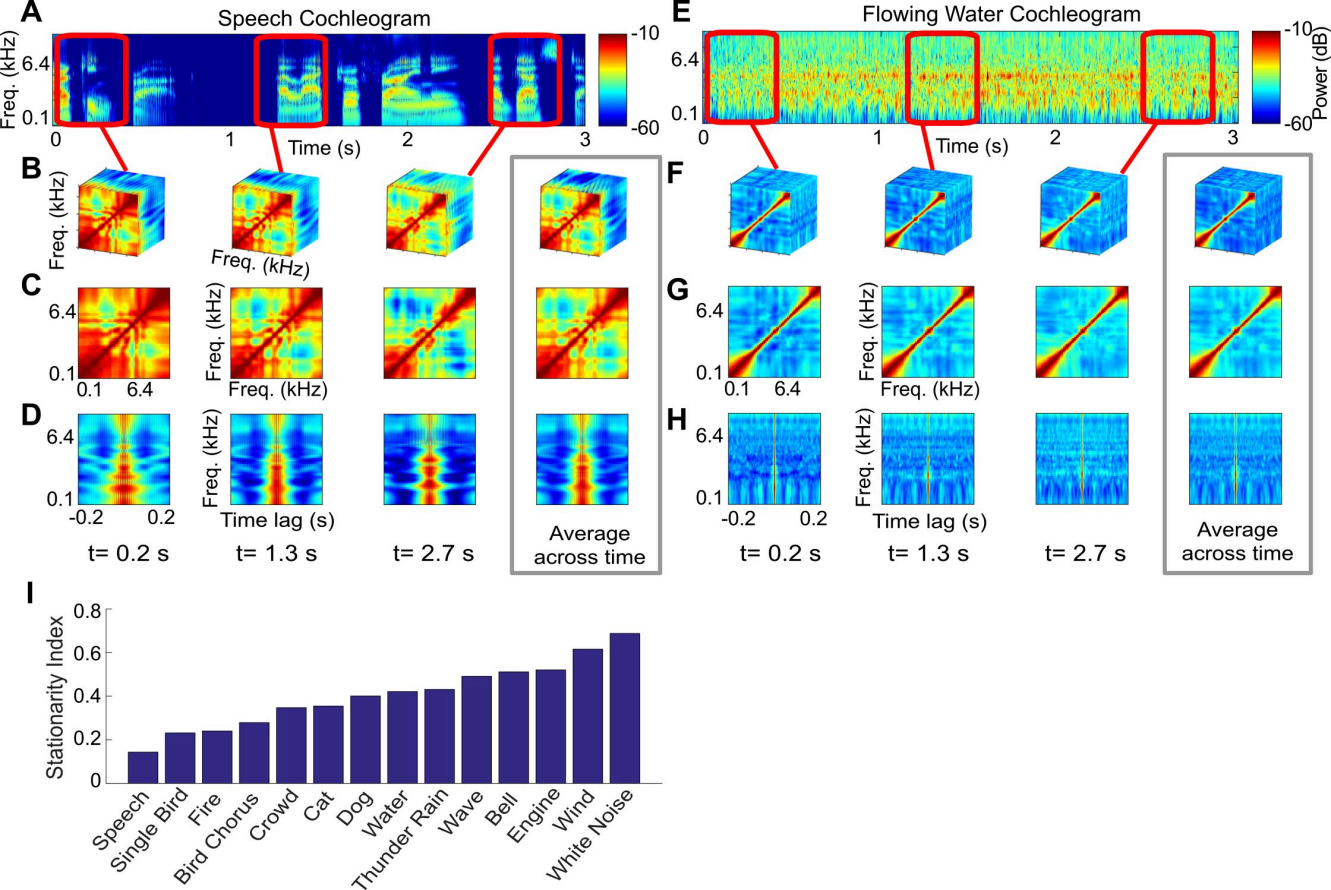
Short-term correlation statistics and stationarity. The short-term correlation statistics are estimated by computing the spectro-temporal correlation matrix using a moving sliding window. The procedure is shown for an excerpt of (A–D) speech and (E–H) water (additional examples in S1–S4 Movies). The sliding window (400 ms for these examples) is varied continuously over all time points but is shown for three select time points for this example. The short-term statistics are also shown for the spectral and temporal correlation decompositions. Note that for speech, the correlations change dynamically from moment to moment and differ from the time-averaged correlations (gray panel), indicating nonstationary structure. By comparison, the time-varying correlations for water resemble the time-averaged correlations (gray panel), indicating more stationarity. (I) SIs for the 13 categories and white noise. Speech has the lowest stationarity values, while white noise is the most stationary sound. Principal components derived for the short-term spectral and temporal correlations of all natural sounds in the database are shown in S8 Fig. Figure data and related code are available from http://dx.doi.org/10.6080/K03X84V3. Freq., frequency; SI, stationarity index.

The short-term correlation decomposition of a speech and a flowing water sound excerpt illustrate extreme differences in the time-varying correlation statistics (Fig 7; A–D for speech; E–H for water; see additional examples shown as S1–S4 Movies). Speech is highly nonstationary at the timescale shown (400-ms moving window) and the correlation structure (spectro-temporal, spectral, and temporal correlations) varies considerably from one instant in time to the next. Such nonstationary spectro-temporal correlation structure is, in part, due to the differences between periods of speech and silence. However, even within speech periods, the correlation structure can vary and can be quite distinct for each word and differ from the average correlation structure (Fig 7B–7D; average shown in gray boundary). These differences likely reflect the range of articulatory mechanisms involved during speech production and the rapidly changing phonemes, formants, and pitch. In sharp contrast to speech, water sounds are relatively stationary. For an example sound, the correlation statistics at each instant in time are relatively consistent and closely resemble the time-averaged correlation (Fig 7F–7H; average shown in gray boundary). Regardless of the time segment we examine, spectral correlations are diagonalized, indicating that only neighboring channels have similar envelopes, while temporal correlations are relatively fast, with similar fast time constants across all frequencies.

To quantify the degree of stationarity (or lack of), we developed a stationarity index (SI; see Materials and Methods) with a value of 1 indicating perfect stationarity and a value of 0 indicating that the sound is highly nonstationary. Although the results are quite varied, we note several trends. First, except for fire sounds, environmental sounds (including running water, waves, thunder and wind) tend to have the highest average SI (SI = 0.49 ± 0.04; mean ± SE). This might be expected, given that such environmental sounds typically consist of mixtures of randomly arriving sound elements (e.g., water droplets, air bubbles, etc.) and have been shown to be perceptually well-described by average statistics [2,8]. By comparison, vocalized sounds have a somewhat lower nonstationary index (SI = 0.28 ± 0.04; mean ± SE) at the analysis timescales employed. This is expected, given that vocalizations transition from periods of silence and vocalizations at timescales of just a few Hz [31], and even within vocalization segments, the correlation structure can vary dynamically from moment to moment (e.g., Fig 7). In the classification analysis that follows, we aim to describe this nonstationary structure and determine to what extent ignoring nonstationary impairs sound categorization.

### Decoding neural and sound-correlation statistics to categorize sounds

Given that the neural correlation statistics are modulated by the correlation structure in sounds and that natural sounds have highly varied correlation statistics, we next tested whether these statistics could directly contribute to sound category identification. Here, we aim to quantify the specific contributions of spectral and temporal correlations. While both spectral and temporal cues can contribute to a variety of perceptual phenomena, they often do so differentially. For instance, speech recognition can be performed with low spectral resolution so long as temporal details are preserved [33], whereas music perception requires much finer spectral resolution [34]. Thus, it is plausible that one of the two dimensions (temporal or spectral) could be more informative for specific sound categories or for the sound category identification task as a whole.

#### Decoding neural correlation statistics in a three-category identification task

**Fig 8:**
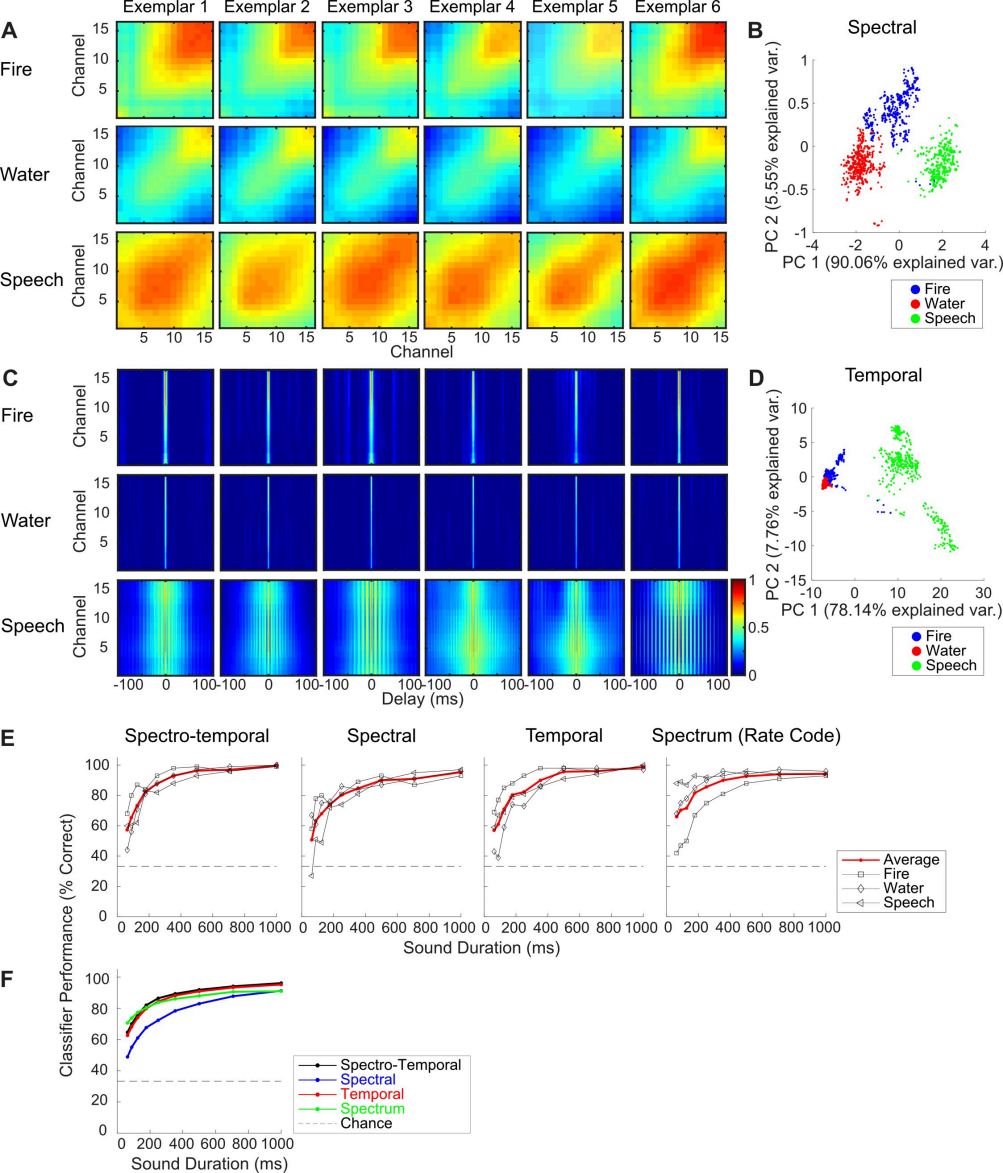
Using neural ensemble correlation statistics to categorize sounds in a three-category identification task. The sound categories delivered included fire, water, and speech, with six exemplars per category. As shown for a representative IC penetration site, spectral (A) and temporal (C) neural correlations (100 observations × 6 exemplars × 3 categories; see Materials and Methods) show distinct structures across sound categories but are similar within a category. Projections of the neural correlations onto the first two principal components show that spectral (B) and temporal (D) correlation form distinct clusters for each of the three sound categories. (E) Single-response trial classification results for this penetration site. In all cases, classifier performance improves with sound duration approaching near 100% for the full sound duration (1 sec). (F) Average performance across IC penetration sites (*N* = 11; *N* = 3 and 8 from two animals). The spectro-temporal classifier has the highest performance, with an average performance of 96% correct classification for 1 s duration. Figure data and related code are available from http://dx.doi.org/10.6080/K03X84V3. IC, inferior colliculus; PC, principal component.

We first developed a three-sound–category neural recording paradigm to test whether neural correlation statistics in the IC can contribute to categorizing sounds. The stimulus-driven spectral and temporal correlations from an example IC penetration site for sounds from three categories (fire, water, and speech; see Materials and Methods) are shown in Fig 8A and 8C. Within a sound category, spectral and temporal correlations are very similar across different sound exemplars, while across sound categories, correlation matrices are diverse and show distinct structures. In this example site (frequency response areas approximately 3–6 kHz), neural responses to water sounds have relatively restricted spectral correlations and fast temporal correlations. Spectral correlations are more extensive for the fire sounds, with the stronger correlations localized at high-frequency channels, although temporal correlations are succinct. For speech sounds, spectral correlations are widespread, and temporal correlations are relatively slow. Such diverse neural correlation statistics indicate that they could be used for sound categorizations.

In a three-category identification task, we applied principal component analysis (PCA) to the correlation feature vectors (a low-dimensional representation of the neural correlations) and fitted a naïve Bayes classifier (see Materials and Methods). After training the model with the responses of five exemplars per sound category, we used the Bayes classifier to categorize single-response trials obtained from the remaining exemplar (leave-one-out cross validation; see Materials and Methods). Fig 8B and 8D show the projections of the spectral and temporal correlations for the training data set onto the first two principal components from the same penetration site. With two principal components, most of the response variance is explained (95.6% for spectral and 85.9% for temporal), and the three categories are highly clustered, suggesting that neural correlations for the three sound categories are distinct from each other.

The neural correlation classifier accurately categorized the sounds in the three-category identification task. For the example recording site (Fig 8E, same site as Fig 8A–8D), all classifiers (spectro-temporal, spectral, and temporal) show increasing performance with sound duration, and the categorization accuracy approached 100% when the sound is 1 s. The spectrum (rate code) classifier also achieves high accuracy in this categorization task (94% for 1 s duration). Averaging across all penetration sites, neural classification performance improves with increasing sound duration (Fig 8F). For 1 s sound duration, the spectro-temporal classifier is above chance and achieves on average a 96% maximum accuracy (*t* test, *p* < 0.01), followed by the temporal classifier (95%, *t* test, *p* < 0.01) and spectral (91%, *t* test, *p* < 0.01). The spectrum (rate code) classifiers also achieve high performance for 1 s duration (91%, above chance, *t* test, *p* < 0.01). These results suggest that for the chosen categories and sounds, neural correlations are sufficiently stable (within category) and distinct (across categories) to enable identification of sound categories.

#### Decoding sound-correlation statistics to categorize sounds

To further evaluate the role of correlations for sound category identification, we next developed a statistical classifier applied to the short-term correlation statistics of natural sounds (Fig 7) and evaluated the model’s performance in a 13-category identification task. After computing the time-varying correlations for purely spectral, purely temporal, or spectro-temporal correlations (Fig 7), we again reduce the dimensionality of the features using PCA (S8 Fig). We then fit the low-dimensional representation of the correlations using an axis-aligned Gaussian mixture model (GMM) for each sound category (see Materials and Methods). After training, we classify test sounds by comparing the posterior probability of the sounds under the mixture models of each category. By limiting the short-term correlations to spectral (Fig 7C and 7G), temporal (Fig 7D and 7H), or spectro-temporal (Fig 7B and 7F), we can measure how each of these acoustic dimensions contributes to categorizing sounds (see Materials and Methods).

**Fig 9:**
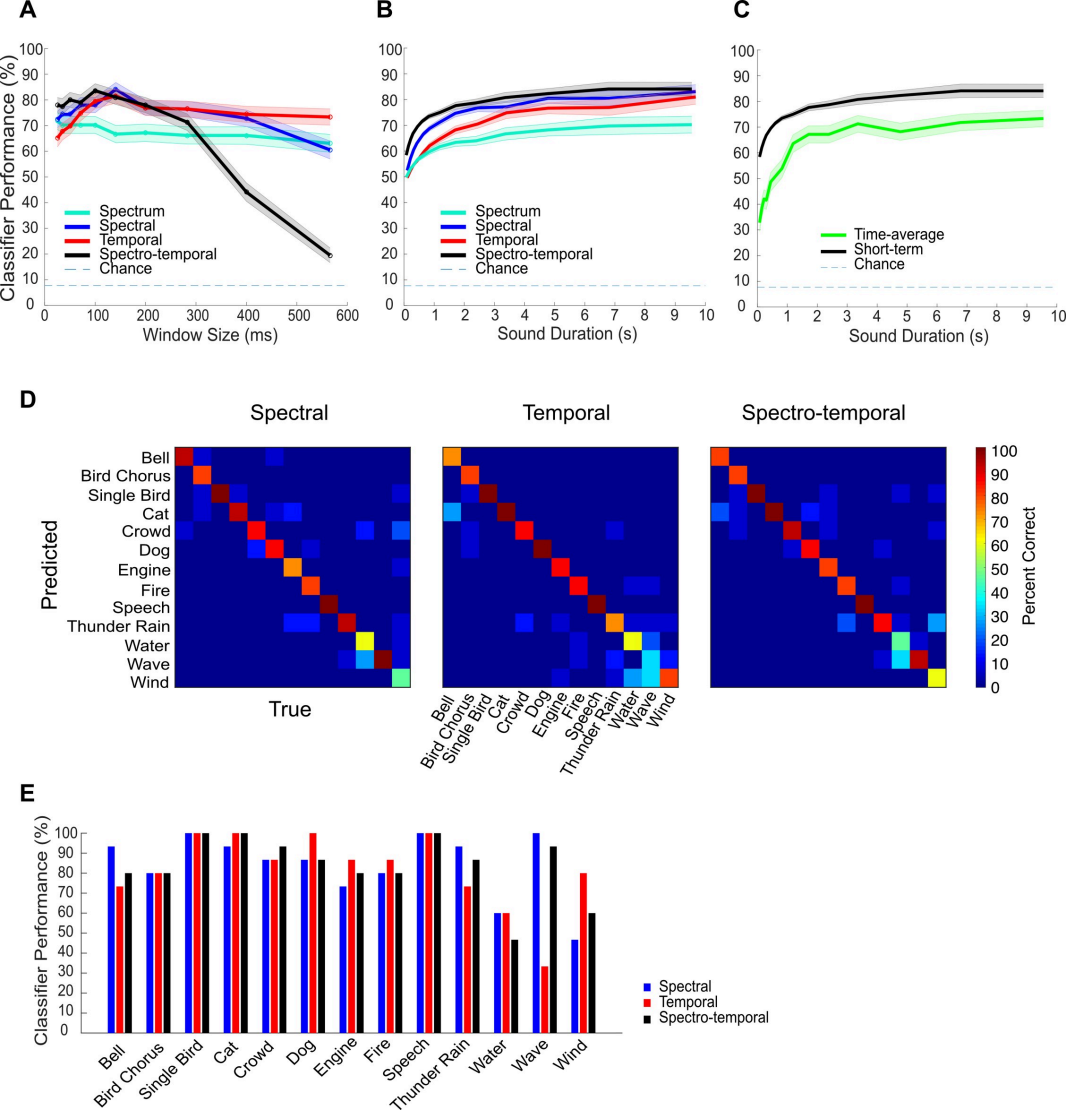
Using short-term correlation statistics to categorize sounds in a 13-category identification task. A cross-validated Bayesian classifier is applied to the sound short-term correlation statistics (spectral, temporal, and spectro-temporal) to identify the category of each of the test sounds (see Materials and Methods). (A) Both the spectral and temporal correlation classifiers had an optimal temporal resolution of 144 ms (i.e., short-term analysis window size). The optimal resolution of the spectro-temporal correlation classifier, by comparison, is slightly higher (100 ms). For reference, the performance of a spectrum-based classifier is largely independent of the sound resolution used. (B) For all three correlation classifiers and the spectrum classifier control, the performance improves with the sound duration. The spectro-temporal correlation classifier performance improved with sound duration at the fastest rate, while temporal correlations had the slowest rate of improvement. The correlation-based classifiers outperformed the spectrum-based classifier performance in all instances. (C) The short-term spectro-temporal classifier outperforms the time-averaged classifier, indicating that nonstationary structure improves performance. (D) Confusion matrices for the three correlation-based classifiers for 10 s sound durations. (E) Performance for the three classifiers shown as a function of sound category (measured at the optimal resolution and at 10 s sound duration). Figure data and related code are available from http://dx.doi.org/10.6080/K03X84V3.

We optimized the model and classifier for each task separately using multiple temporal resolutions (*τ*_*W*_ = 25–566 ms; Fig 9A; optimized at 10 s duration). The optimal window resolution for both the temporal and spectral classifiers is 141 ms, while the optimal resolution for the joint spectro-temporal classifier is slightly faster (100 ms). Classification performance is above chance for all temporal resolutions tested (Fig 9A; chance performance = 7.69%; *p* < 0.01, *t* test, Bonferroni correction), and classification performance improves with increasing sound duration (Fig 9B). Furthermore, all three classifiers achieve similar maximum performance at approximately 10 s (spectro-temporal = 84%, spectral = 83%, temporal = 81%; spectral versus temporal: *p* = 0.60; spectral versus spectro-temporal: *p* = 0.79; temporal versus spectro-temporal: *p* = 0.42; two-sample *t* test). This indicates that both spectral and temporal correlations contribute roughly equally to the sound identification task for the full sound duration. However, the spectral classifier performance increases at a faster rate than the temporal classifier (reaching 90% of maximum in 1.7 versus 3.0 s; Fig 9B), indicating that evidence about the sound category may be accumulated more efficiently using spectral correlations. The joint spectro-temporal classifier improves at an even faster rate (reaching 90% of its maximum in 1.2 s). Finally, ignoring the nonstationary structure of the spectro-temporal correlation by averaging the statistics over time reduced the sound category identification performance by approximately 25% (Fig 9C). Thus, the time-varying statistical structure of the sounds can contribute to more accurate sound categorization.

As with the neural correlations, we find distinct differences between the performance of the spectral and temporal classifiers for the individual sound categories (Fig 9D and 9E). Certain sounds such as waves perform substantially better for the spectral classifier (spectral = 100% versus temporal = 33%). Other sounds, such as wind, exhibit higher performance with the temporal classifier (spectral = 47% versus temporal = 80%). Thus, although on average, the performance of the spectral and temporal classifiers is comparable, performance of certain sound categories appears to be dominated by one of the two sets of features (Fig 9E).

As a control, we also compared the performance of the correlation-based classifier against the performance for a classification strategy utilizing the sound spectrum (Materials and Methods). Although useful for discriminating sounds, the sound spectrum provides little information about the sound identity in most instances [2] and is strongly affected by the sound source direction [6] and physical characteristics of the room or acoustic environment (e.g., size, shape, and boundary materials) [35]. Thus, it is possible that the sound-correlation structure is a more invariant cue for the categorization task. Unlike the correlation-based classifiers, performance of the spectrum-based classifier does not depend on the temporal resolution of the analysis (Fig 9A), indicating that it does not benefit from nonstationary time-dependent information. Furthermore, the overall performance of the spectrum classifier is lower than the either of the three correlation-based classifiers (Fig 9B). This supports the hypothesis that the stimulus correlation structure is a more informative and invariant cue for the categorization task.

**Fig 10:**
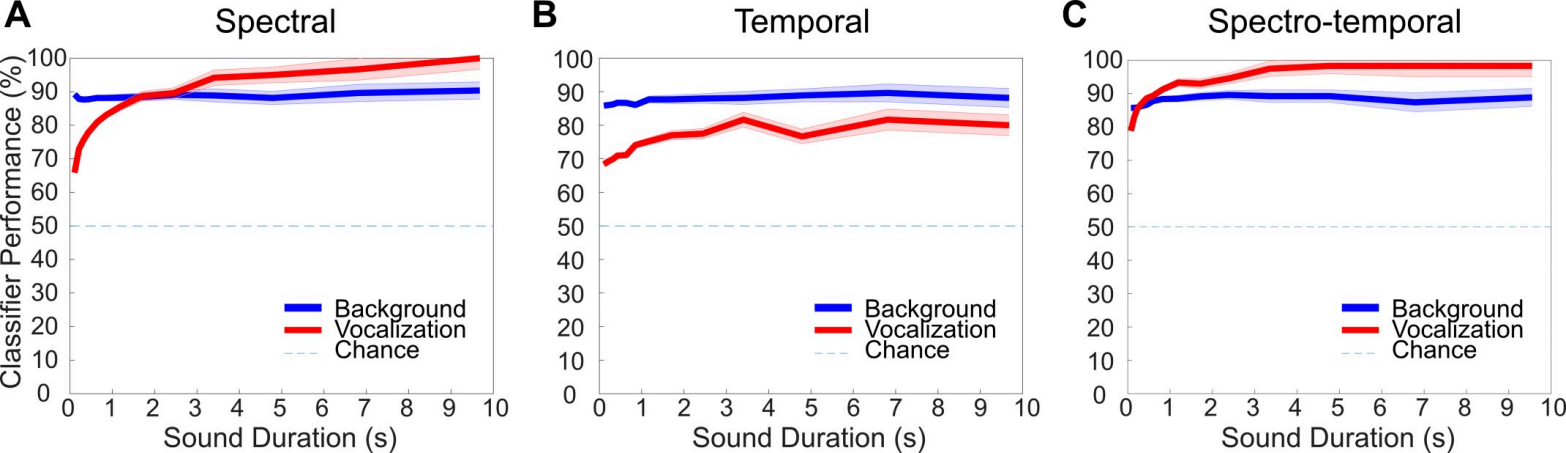
Model classification performance in a two-category identification task. The classification task requires that the model distinguish vocalization from background sound categories. For all three classifiers, the overall performance is consistently high and improved with increasing sound duration. Vocalization classification accuracy is highest for the spectro-temporal classifier (C) and shows a nearly identical trend for the spectral classifier (A). The performance of the temporal classifier, however, is approximately 20% lower. For background sounds, classification accuracy does not improve over time and is consistently high (approximately 90%) for all three classifiers. Figure data and related code are available from http://dx.doi.org/10.6080/K03X84V3.

Finally, we evaluated the performance of the classifier in a two-alternative forced choice task in which we required the classifier to distinguish vocalization and background sound categories. The model performance is consistently high, with accuracy rates for the spectro-temporal classifier approaching 90% for background sounds and nearly 100% for vocalizations (Fig 10). Interestingly, the performance for identifying vocalizations improves with increasing sound duration, while the performance for background sounds remains constant for all three classifiers. Since background sounds are more stationary, their statistics can be assessed quickly in this task by the classifier. Vocalizations, on the other hand, are nonstationary over longer timescales and have epochs of silence that may require the classifier to accumulate evidence over longer time. Additionally, the classification accuracy of background sounds is comparable (approximately 90%) for spectral and temporal features. By comparison, vocalizations are more accurately classified with spectral compared to temporal correlations (*p* < 0.01, *t* test with Bonferroni correction), and the spectral correlations alone appear to account for most of the spectro-temporal classifier performance.

## Discussion

Here, we have demonstrated that natural sounds can have highly structured spectro-temporal correlations and can induce highly structured correlations between neural ensembles in the auditory midbrain. Unlike noise correlations, which are largely unstructured, stimulus-driven neural correlations in both time and frequency (or space) are highly informative and can convey critical information about the sound or category identity, with evidence accumulation times on the order of a few seconds.

### Using sound-driven correlation statistics for recognition

Previous work has emphasized the distinct roles of noise-driven and stimulus-driven correlations in limiting the information capacity of neural ensembles. Noise correlations, which are the result of coordinated firing not related to the stimulus structure, are thought to limit the encoding of sensory information [20,21]. At the same time, theories of efficient coding suggest that neural ensembles should minimize the amount of stimulus-driven correlated firing to limit redundancies in the neural code [11,12,19]. Here, we find that while stimulus-driven correlations in the IC are highly structured, noise correlations are largely unstructured. Noise correlations are localized primarily to nearby recording channels and brief time epochs, and they do not vary systematically with the sound. These findings thus provide an alternative and, perhaps, underappreciated viewpoint: correlations in neuron ensembles can convey critical information about the stimulus ensemble itself. Rather than a redundant structure in the feature-based representation that should be removed [12], sound correlation can be viewed as high-level acoustic features or cues that are highly informative about particular sound or category and that, as demonstrated, drive correlated activity in midlevel auditory structures. Thus, rather than simply discarding correlated neural activity, downstream cortical circuits could transform and utilize such signals for the purpose of recognition.

Indeed, recent studies on sound textures confirm that correlation structure is a critical cue required to create realistic impressions of sounds [2], and there is growing evidence from neural recordings that neural correlations contribute to signal coding and decision-making [36–38]. Our findings extend these views by demonstrating that stimulus-driven correlations between neural ensembles in a midlevel auditory structure can directly vary with and contain information about sound identity and that sound categories have unique correlation statistics that may promote or enhance sound categorization. The measured neural correlations are only mildly affected by manipulations in the sound spectrum (Fig 4), which often arise through head-related filtering [6] and room characteristics [35]. Future studies will need to parse out the possible limits to such a code, given natural spectral variability in the environment and the fact that temporal structure of sounds can be distorted by reverberation [39].

### Biological plausibility

While our results demonstrate that neural correlations in the IC are highly structured, how and whether such information is used by higher brain regions needs further exploration. One possible mechanism for computing correlations between neurons has been previously considered for pitch detection: the frequency-coincidence detection network [23,24]. The key proposal of this network is that neurons encoding different frequencies project onto the same downstream neurons that then detect coincident firing. Given that anatomical connections within and beyond the IC can span a broad range of frequencies, the central auditory system anatomy allows for such a possibility. Many projection neurons in the IC have long-range collaterals that integrate across different frequencies and send their outputs to the auditory thalamus [40]. Thalamocortical and intracortical connections, although frequency-specific, can also extend across several octaves [41,42]. This pattern of connectivity has the capacity to integrate information across frequency channels and may subserve a coincidence-like operation between inputs of different frequency. Such coincidence detection could allow downstream neurons to compute spectral correlations not just for pitch detection but for sound categorization, as well.

### Contribution of spectral and temporal correlations

A key question brought up by the model and neural data is the extent to which spectral and temporal correlations individually contribute to sound recognition. Although some differences could be observed for specific sounds or categories, both dimensions appear to contribute roughly equally. Performance of the model classifier is only marginally better when the two dimensions are combined, and the optimal neural classifier used roughly equal proportions of spectral and temporal correlations (S9 Fig). Thus, both dimensions contain sound-related information that can contribute to recognition.

Insofar as neural mechanisms for computing the sound-correlation statistics go, spectral models have broader biological support. Spectral correlations could be computed using coincidence detection, in which two neurons tuned to distinct frequencies converge multiplicatively on downstream neurons. Such spectral convergence is widespread in auditory system anatomy, and the required multiplicative interactions have been previously described [43,44]. On the other hand, computing temporal correlations requires coincidence detection at different times, which can be achieved by delay lines or feedback loops. Such mechanisms, however, are more speculative and lack strong anatomical or physiologic evidence.

Although some of the neural correlation structure in the IC was accounted for by the model correlation (S5 Fig), neural correlations tend to be more complex in the IC. For one, correlated firing in IC is shaped by its anatomy because correlated firing is reduced with increasing distance across frequency lamina but not within a lamina [19,45]. Furthermore, in contrast to peripheral auditory filters, which are selective for frequency content, IC neurons are also selective for temporal and spectral modulations that are critical elements of all natural sounds. This second-order decomposition of a sound into modulation bands further constrains the correlated firing between neurons [19,45]. As such, correlated firing in IC reflects high-level sound correlations that are not strictly selective for frequency content but also for modulation components in natural sounds. Such high-level correlations have been shown to be critical perceptually [2] and, as shown here, have the potential to contribute towards sound recognition.

### Resolution and integration timescales for feature analysis and inference

Sound categorization performance for both the neural data and model improved over the course of 1–2 s and depended strongly on sound duration, similar to human listeners [46,47]. This brings up the question of whether the previously observed perceptual integration times in human observers should be attributed to a slow central neural integrator. Rather than computing average statistics about a sound over long timescales, it is plausible that sound statistics themselves are integrated and estimated by the auditory system at relatively short timescales analogous to the optimal integration window resolution of our model (approximately 150 ms). The long timescales of a few seconds required to make perceptual decisions may instead reflect a statistical evidence accumulation process, as previously proposed for cortical areas involved in decision-making [48].

In modeling correlations in natural sounds, we find that the times required to accumulate statistical information about sound categories are roughly an order of magnitude larger than the optimal temporal window for calculating the correlations (approximately 100–150 ms). This is consistent with a temporal-resolution–integration paradox previously observed for neural discrimination of sounds [49]. Here, the correlations are estimated using an optimal time window of approximately 100 ms while evidence accumulates over these consecutive windows, achieving 90% of the maximum performance in the time course of a few seconds (evidence accumulation time). From a neural coding perspective, the temporal resolution can be viewed as the time window over which neurons compute the correlations, while the evidence accumulation time represents the time over which a downstream neural population reads the correlations to accumulate evidence. Our estimated timescales are substantially longer than those previously reported for neural discrimination of sounds in auditory cortex (approximately 10 ms and approximately 500 ms), which likely reflects differences in the acoustic features and task [49,50]. The timescales for auditory cortex in these previous studies were optimized for discrimination of pairs of sounds based on spike-train distance measures at a single-neuron level. By comparison, here we use high-order correlation statistics of neuron ensembles as the primary feature for categorizing many more sounds.

## Conclusion

The results show that the correlation structure of natural sounds shows reliable differences between categories. Surprisingly, this acoustic structure is mirrored in the correlation of IC neural ensembles, and such neural correlations can be decoded to accurate recognize individual sounds or categories. The combined results suggest that correlated firing in the auditory system may play an important role not just in pitch perception or localization but in sound recognition more generally.

## Materials and methods

### Ethics statement

All animals in the study were handled according to approved procedures by the University of Connecticut Animal Care and Use Committee (protocol A18-056) and in accordance with National Institutes of Health and the American Veterinary Medical Association guidelines.

### Animal experimental procedures

Multichannel neural recordings are performed in the auditory midbrain (IC) of unanesthetized female Dutch Belted rabbits (*N* = 4; 1.5–2.5 kg). Rabbits are chosen for these experiments because their hearing range is comparable to that of humans and they sit still for extended periods of time, which enables us to record from different brain locations daily over a period of several months.

### Surgery

All surgical procedures are performed using aseptic techniques. Surgical procedures are carried out in two phases with a recovery and acclimation period between procedures. For both procedures, rabbits are initially sedated with acepromazine and a surgical state of anesthesia achieved via delivery of isoflurane (1%–3%) and oxygen (approximately 1 L/min). In the first procedure, the skin and muscle overlying the dorsal surface of the skull are retracted, exposing the sagittal suture between bregma and posterior to lambda. Stainless steel screws (0–80) and dental acrylic are used to affix a brass restraint bar, oriented rostrocaudally and to the left of the sagittal suture. Dental acrylic is then used to form a dam on the exposed skull on the right hemisphere between lambda and bregma. Next, custom-fitted ear molds are fabricated for each ear. A small cotton ball is inserted to block the external auditory meatus and a medical-grade polyelastomeric impression compound poured into the ear canal. After approximately 5 minutes, the hardened impression compound is removed. The ear impression mold is subsequently used to build a cast from which custom ear molds are fabricated.

Following the first surgery and a 5-day recovery period, the animal is acclimated over a period of 1–2 weeks to sit still with the head restrained. During this period, the animal is also gradually exposed to sounds through the custom-fitted ear molds. Once the animal is capable of sitting still during sound delivery, the second surgical procedure is performed. The animal is again anesthetized as described above, and an opening (approximately 4 × 4 mm) is made on the right hemisphere within the dental acrylic dam and centered approximately 10–12 mm posterior to bregma. At this point, the exposed brain area is sterilized with chlorhexidine solution, and medical-grade polyelastomeric compound is poured into the acrylic dam to seal the exposed region.

### Sound delivery and calibration

Sounds are delivered to both ears via dynamic speakers (Beyer Dynamic DT 770 drivers; beyerdynamic, Heilbronn, Germany) in a custom housing and custom-fitted ear molds obtained as described above. The molds are fitted with a sound delivery tube (2.75 mm inner diameter) that is connected to the dynamic speaker housing, forming a closed audio system. Calibration consisted of delivering a 10-s–long chirp signal at 98 kHz sampling rate via TDT RX6 (Tucker-Davis Technologies, Alachua, FL, USA) and measuring the audio signal with a B&K calibration microphone (Brüel and Kjær, Nærum, Denmark) and probe tube placed approximately 5 mm from the tympanum. The measured signal is used to derive the sound-system impulse response (via Wiener filter approach; combined speaker driver and tube), and an inverse filter finite impulse response is then derived. Subsequently, all sounds delivered to the animal are passed via the inverse filter, which is implemented in real time using a TDT RX6 (Tucker-Davis Technologies) at 98 kHz sampling rate. The sound delivery system has a flat transfer function and linear phase between 0.1–24 kHz (flat to within approximately ±3 dB).

At each penetration site, we first delivered a pseudorandom sequence of tone pips (50 ms duration, 5 ms cosine-squared ramp, 300 ms intertone interval) covering multiple frequencies (0.1–16 kHz) and sound pressure levels (SPLs) (5–75 dB SPLs). These tone-pip sequences are used to measure frequency response areas, which allows us to estimate the frequency selectivity of each recording site (different channels).

#### Sound paradigm 1

Next, across *N* = 13 recording locations (*N* = 4 and 9 across two animals), we delivered a sequence of five environmental noises with distinct structural properties to determine whether the ensemble activity of the auditory midbrain reflected frequency-dependent correlation statistics present in the sounds and to determine whether such neural population statistics can be used identify the individual sounds. Each sound is 3 s duration and is delivered in a block-randomized fashion with a 100-ms interstimulus interval between sounds. To avoid broadband transients, the sounds have b-spline onset and offset ramps (20 ms rise–decay time) and are delivered at an RMS SPL of 65 dB RMS SPL; 87 dB peak SPL over all sounds. Each recording had 5 sound conditions in which each sound had at least 18 trials (range 18–39), with a 3-s duration for each trial. The sounds included crackling fire, bird chorus, outdoor crowd, running water, and a rattling snake sound. These sounds each contain unique time-frequency correlation statistics, allowing us to test and quantify whether such statistics are potentially encoded by the auditory midbrain and ultimately represented in the neural ensemble activity. For instance, the water sounds have minimal across-frequency channel correlation because the air bubbles and droplets responsible for this sound are relatively narrow-band and occur randomly in time, thus activating frequency channels independently [8]. Sounds such as crackling fire, by comparison, have strong frequency-dependent correlations because of crackling embers, which produced brief impulsive “pops” that span multiple frequency channels simultaneously. The temporal correlations of these five sounds are also quite varied. For instance, the water sound has a very brief impulsive correlation structure lasting just a few milliseconds, whereas the bird chorus and the outdoor crowd, which contains multispeaker speech babble, have a broader and slower temporal correlation function. The rattling snake sound, by comparison, had strong periodic correlations at approximately 20 Hz.

#### Sound paradigm 2

In *N* = 11 recording locations (*N* = 3 and 8 across two animals), we also delivered the above sounds (paradigm 1) using variants with matched power spectrum. This was done by synthetically equalizing the sounds so that all sounds have 1/f power spectrum. This manipulation removes all spectral cues but preserves many of the fine structure and high-order modulation cues in the original sounds. Furthermore, the 1/f spectrum is chosen as opposed to a flat spectrum because it provides roughly uniform activation across the tonotopic axis (equal power per octave). The paradigm allows us to dissociate and examine how spectral and high-level correlation cues contribute to the neural population activity. Although all five sounds have identical power spectra, they can still be easily identified. This seems to indicate that consistent with prior findings [2], the power spectrum is not critical for sound identification.

To generate the 1/f equalized sounds, we first estimated the sound magnitude spectrum of each sound, *S*(*f*), using a Welch average periodogram (b-spline window; frequency resolution = 10 Hz; 40 dB sidelobe error). The spectrum of each sound was then used to generate a zero-phase inversion filter with transfer function of the form

$$H(f)=C\cdot S{\left(f\right)}^{-1}f^{-1},$$

where the *C* is a gain normalization constant required to assure that the sounds have identical SPLs (65 dB RMS; matched to the SPL for paradigm 1). Each sound was next filtered with *H*(*f*), thus producing a synthetic variant with identical 1/f spectrum (see S7 Fig). To minimize the effects of adaptation, the equalized sounds were interleaved with the original sounds in paradigm 1 (as controls) and delivered in block-randomized fashion with a 100-ms interstimulus interval between sounds. Sounds were delivered for *N* = 20 trials in all recording sites but one, which had *N* = 14 trials.

#### Sound paradigm 3

Finally, in *N* = 11 recording locations (across two animals, *N* = 3 and 8), we tested whether neural ensemble statistics in the IC can contribute to the identification of acoustic categories. We delivered a sequence of sounds selected from three acoustic categories (fire, water, and speech), each containing multiple exemplars (6 exemplars per category; 18 sounds total; see sound spectrum in S7 Fig). These sounds represent a subset of the sounds used subsequently for the neural model classifier (Materials and Methods, see below; S2 Table). Sounds were delivered at 65 dB SPL in block-randomized order. A minimum of 10 sound trials (maximum = 20) were delivered at each recording location.

### Electrophysiology

Multichannel acute neural recording silicon probes (Neuronexus 10 mm probe; 16 linear spaced recording sites with 150 μm separation and 177 μm^2^ contact area or 64-channel polytrodes with 60 μm separation and 177 μm^2^ contact area [used for paradigms 2 and 3]; site impedance approximately 1–3 *M*Ω) are used to record neural activity from the IC of unanesthetized rabbits. In the case of the 64-channel polytrodes, we selected a subset of equally spaced 16 channels for analysis in order to ensure that the data format and sampling of the IC were comparable to that of the 16-channel linear probes. We recorded neural data from *N* = 33 penetration sites in 4 rabbits. Viable recording sites were selected by requiring that the neural activity had stable response magnitudes for a minimum of 15 trials for paradigm 1 and 10 trials for paradigms 2 and 3. These requirements were imposed to assure that the correlation-based classifier had a sufficient number of trials that could be used for model generation and validation step (described subsequently). In addition, we required that the recording locations had a well-defined tonotopic gradient and were consistent with the central nucleus of the IC [51–53]. This selection resulted in *N* = 13 penetration sites for paradigm 1 and *N* = 11 sites for paradigms 2 and 3. Since there are 16 recording channels for each penetration site, data are obtained from a total of 16 × 24 = 384 recording sites within the IC.

Prior to recording, the polyelastomeric compound is removed from the craniotomy, and lidocaine is applied topically to the exposed cortical tissue. The area is then flushed with sterile saline and the acute recording probe inserted at approximately 12–13 mm posterior to bregma. If necessary, a sterile hypodermic needle is used to nick the dura to allow the electrode to penetrate the neural tissue. An LS6000 microdrive (Burleigh EXFO, Quebec, Quebec, Canada) is used to insert the neural probe to a depth of approximately 7.5–9.5 mm relative to the cortical surface, where, at this penetration depth, most or all of the recording electrodes are situated in the IC and have clear responses to brief bursts of broadband noise or tones. Neural activity is acquired continuously at sampling rate of 12 kHz using a PZ2 preamplifier and RZ2 real time processor (Tucker-Davis Technologies).

The sampled extracellularly recorded neural signals are analyzed offline using an analog representation of multiunit activity (analog multiunit activity [aMUA]) [54]. We use this multiunit representation for several reasons. First, neighboring neurons in the IC have similar spectro-temporal preferences and strong correlated firing, restricted to a relatively small neighborhood of approximately 100 μm, and similar best frequencies [19,45]. The electrodes used in the study have identical electrical properties (impedance, contact area) and a similar recording radius (estimated approximately 100 μm) to those we have used previously [45] and thus isolate neural activity on each channel from mostly independent neural ensembles. Second, unlike single-unit activity, the local aMUA signals can be obtained from all 16 recording sites simultaneously, which allows us to study how local populations in the IC represent acoustic information along the tonotopic axis. We did not use single-unit analysis for this study because although we are able to spike-sort the data and identify single neurons, on average, we are only able to isolate at most approximately 3 neurons per recording session across the entire electrode array [3,19]. In addition, although we considered using more conventional measures of multiunit activity obtained by thresholding, past work suggests that aMUA signals capture the structure of population activity with substantially less noise [54]. Here, when we compare the correlations between electrodes measured with thresholded MUA (thresholding criteria, 2× SDs above the noise floor) and aMUA, we find that the correlation patterns are very similar but that the correlations with thresholded MUA are weaker and noisier (S10 Fig). In this experimental setting, aMUA signals are thus better suited than single-unit or thresholded MUA to quantify correlations across a large tonotopic spans and to study classification performance with large populations.

From each of the recorded neural traces, aMUA is measured by first extracting the envelope of the recorded voltage signal within the prominent frequency band occupied by action potentials spanning frequencies 325 and 3,000 Hz (b-spline filter, 125 Hz transition width, 60 dB attenuation) [55]. The bandpass-filtered voltage signal is next full-wave rectified and low-pass–filtered with a cutoff frequency of 475 Hz (b-spline filter, transition width of 125 Hz, and stopband attenuation of 60 dB) because neurons in the auditory midbrain typically do not phase-lock to envelopes beyond approximately 500 Hz [53]. The resulting envelope signal is then downsampled to 2 kHz. Such neural envelope signals capture the synchronized activity and the changing dynamics of the local neural population with each recording array in both time and frequency domains [54]. For each recording channel, an analog raster is generated that consists of the aMUA response over time and across trials.

### Neural ensemble stimulus-driven correlation

For each recording penetration, we first estimate the stimulus-driven correlations of the neural ensemble across the 16 recording channels directly from the measured aMUA signals. The procedure consists of a modified windowed short-term correlation [32] analysis between recording sites, in which the correlations are “shuffled” across response trials [19,56]. The windowed correlation approach allows us to localize the correlation function in time, whereas the shuffling procedure is used to remove neural variability or noise from the correlation measurements [56]. Thus, the proposed shuffled windowed correlation allows us to isolate stimulus-driven correlation between recording sites independently of noise-driven correlations. The windowed shuffled cross-correlation between the *k*-th and *l*-th recording site is computed as the mean pairwise correlations between different trials of the aMUA envelope:

$$\Phi_{kl}^{stim}\left(t,\tau \right)=\Phi_{kl}^{shuffled}\left(t,\tau \right)=\frac{1}{N\left(N-1\right)}\overset{N}{\underset{m=1}{\sum }}\underset{n\neq m}{\sum }\phi_{kl,mn}\left(t,\tau \right)$$

where *N* is the number of trials in each recording channel (*k* and *l)*. $\phi_{kl,mn}(t,\tau )$ is the windowed cross-correlation between the *m-*th and *n-*th response trials between channels, respectively (*k* versus *l*), where *t* is time and *τ* is the cross-correlation delay:

$$\phi_{kl,mn}(t,\tau )=\int_{-\infty }^{+\infty }r_{k,m}(\gamma )r_{l,n}(\gamma -\tau )W^{2}(t-\gamma )d\gamma ,$$

where *γ* is the time integration variable. Here, *r*_*k*,*m*_(*t*) is the *m*-th response trial (mean removed) from channel *k* and *r*_*l*,*n*_(*t*) is the *n*-th response trial (mean removed) from channel *l*. *W*(*γ*) is the unit amplitude square window centered about *γ* = 0 of duration *T* s (range = 62.5–1,000 ms), which is used to localize the measured signal correlations around the vicinity of the designated time point (*t*). Note that in this formulation, correlations between recording channels (*k* and *l*) are computed for different response trials (*m* and *n*). The above is implemented using a fast-shuffled correlation algorithm according to [56]

$$\Phi_{kl}^{stim}\left(t,\tau \right)=\frac{1}{N\left(N-1\right)}\left(N^{2}\cdot \Phi_{PSTH_{kl}}\left(t,\tau \right)-\overset{N}{\underset{m=1}{\sum }}\phi_{kl,mm}\left(t,\tau \right)\right)$$

where $\Phi_{{PSTH}_{kl}}\left(t,\tau \right)$ is the windowed cross-correlation function for the poststimulus time histograms (PSTHs) between channel *k* and *l*:

$$\Phi_{PSTH_{kl}}(t,\tau )=\int_{-\infty }^{+\infty }PSTH_{k}(\gamma )PSTH_{l}(\gamma -\tau )W^{2}(t-\gamma )d\gamma ,$$

where the PSTHs for the *k*-th and *l*-th channels are

$$PSTH_{k}\left(t\right)=\frac{1}{N}\overset{N}{\underset{m=1}{\sum }}r_{k,m}\left(t\right)$$

$$PSTH_{l}(t)=\frac{1}{N}\overset{N}{\underset{m=1}{\Sigma }}r_{l,m}(t).$$

As previously shown [56], this fast-shuffled correlation algorithm resulted in a marked reduction in the computational time (*N* + 1 correlations compared with *N*(*N* − 1)) for each pair of recording channels. This speedup in the computational time is necessary to bootstrap the data during the model generation and validation applied subsequently (see “Noiseless neural classifier” and “Single-trial neural classifier”).

To remove the influence of the response power on the total correlation measurements, the short-term correlation is normalized as a correlation coefficient. To do so, we note that the neural response correlations are measured using a short-term analysis in which the response statistics of each analysis segment vary dynamically over time. Normalization thus requires that we measured the average localized short-term response variance at each time and delay sample according to

$$\sigma_{k}^{2}\left(t\right)=E\left[var\left[r_{k,m}\left(t\right)W\left(t-\gamma \right)\right]\right],$$

$$\sigma_{l}^{2}(t,\tau )=E\left[var\left[r_{l,n}(t-\tau )W(t-\gamma )\right]\right],$$

where the variance is estimated across time and the expectation is taken by averaging over trials. The normalized stimulus-driven correlation is then obtained as

$${\text{c}}_{kl}^{stim}\left(t,\tau \right)=\frac{\Phi_{kl}^{stim}\left(t,\tau \right)}{\sqrt{\sigma_{k}^{2}\left(t\right)\cdot \sigma_{l}^{2}\left(t,\tau \right)}}.$$

Like a correlation coefficient, this population ensemble correlation function is bounded between −1 and 1. Normally, it is expected that the normalized autocorrelation at zero lag ($c_{kl}^{stim}\left(t,0\right)$) for a single channel is 1. However, because the total response variances ($\sigma_{k}^{2}\left(t\right)$ and $\sigma_{k}^{2}\left(t,\tau \right))$ for neural responses contain both a signal and neural noise (variability) component, the stimulus-driven correlation at zero lag for such a scenario is always <1 (e.g., diagonal terms in Fig 1H).

### Neural ensemble noise correlation

To account for the fact that trial-to-trial variability of the neural activity can potentially affect neural classification performance, we also measured the “noise” correlations across the ensemble. Noise correlations may arise through any stimulus-independent network activity (modulatory, state dependent, etc.) that is unrelated to the physical properties of the acoustic stimulus and are typically estimated by correlating firing rate residuals across repeated presentations of the stimulus [57]. However, because the IC responses can have extremely high precision [19] (milli- down to submillisecond), we developed a modified shuffled correlation procedure in which the noise correlations are obtained by subtracting the shuffled from the unshuffled correlogram. The procedure allows us to measure noise correlations at millisecond and submillisecond timescales relevant to IC. Using the notation above, we first compute the unshuffled correlation obtained by correlating single-trial activity between the *k*-th and *l*-th recording channels:

$$\Phi_{kl}^{unshuffled}\left(t,\tau \right)=\frac{1}{N}\overset{N}{\underset{m=1}{\Sigma }}\phi_{kl,mm}\left(t,\tau \right).$$

The ensemble noise correlation is then obtained as (see S1 Text for proof)

$$\Phi_{kl}^{noise}(t,\tau )=\Phi_{kl}^{unshuffled}(t,\tau )-\Phi_{kl}^{shuffled}(t,\tau ).$$

As for the stimulus-driven correlations, noise correlations are normalized by the total response power

$${\text{c}}_{kl}^{noise}\left(t,\tau \right)=\frac{\Phi_{kl}^{noise}\left(t,\tau \right)}{\sqrt{\sigma_{k}^{2}\left(t\right)\cdot \sigma_{l}^{2}\left(t,\tau \right)}}$$

and are thus strictly bounded between −1 and 1. However, as for the stimulus-driven correlations, the noise autocorrelations at zero lag ${(c}_{kl}^{noise}\left(t,0\right))$ are always <1. However, it is worth noting that the total correlation obtained by combining the stimulus and noise and correlations at zero lag are ${c_{kl}^{total}\left(t,0\right)=c}_{kl}^{stim}\left(t,0\right)+c_{kl}^{noise}\left(t,0\right)=1$. This effect is observed in Fig 1H–1J and all subsequent neural data shown, in which it is noted that the diagonals of the noise and stimulus-driven correlations at zero lag are less than 1 (H and I); however, the combined total correlation is 1 along the diagonal (J).

### Noiseless neural classifier

We first developed a noiseless neural classifier in order to assess whether spatial, temporal, and/or spatiotemporal correlations of the neural ensemble in IC could be used to recognize/categorize sounds. Although technically, the correlations are measured between the neural activity across spatially separated electrode channels (*k* versus *l*), the electrode channels with our recording paradigm are frequency-ordered (Fig 1A). The neural ensemble activity thus reflects spectral correlations between frequency channels, and, in what follows, we describe spatial correlations between recording channels as “spectral correlations.”

The noiseless classifier is implemented using shuffled correlograms of the neural data for both the model generation and the validation steps. The shuffled-correlogram metric removes the noise correlations in the neural data, revealing strictly the stimulus-driven structure [56]. As such, this classifier approximates an upper bound of the classification performance for this specific classification strategy because trial-to-trial variability is not present in the metric. Subsequently, a single-trial classifier is used to assess the real-world performance for this classification strategy.

The noiseless neural classifier is implemented using a cross-validation approach in which half of the data is used for model generation and the second half for model validation. For each recording site, we chose the first half of the aMUA raster for a given sound (1.5 s) as the model and the second half as the validation data. The first and second halves of the data are then swapped, and the procedure is repeated using the first half of the data for validation and the second half for model generation.

Here, we use a naïve Bayes classifier and identify or categorize sounds using a low-dimensional representation of the correlations as features. Classification is carried iteratively for different data segments of the validation data half using a Bayesian approach applied to a low-dimensional representation of the correlation feature vectors, ***c***. The correlation feature vectors ***c*** used for the model are obtained iteratively by measuring aMUA windowed shuffled correlations about randomly selected time points in the modeling data using 500-ms data windows. We then reduce the dimensionality of the correlations by applying principal component analysis to the measured correlation feature vectors ***c*** obtained from the modeling data half. We then fit a naïve Bayes classifier and describe the distribution of principal component scores for each sound or sound category using an axis-aligned multivariate Gaussian distribution (the likelihood). Finally, we select and use the highest-ranked principal components that maximize the classifier performance.

Given an observed response correlation from the validation set, we then use this Bayesian model to evaluate the posterior probability of each sound or sound category. The features fed to the classifier consist of the principal component scores (projections of the feature vectors ***c*** on the highest-ranked model principal components) from the sound’s shuffled spectral, temporal, or spectro-temporal correlation. Since we are interested in how categorization performance changes with the sound duration (varied from 62.5 ms to 1,000 ms in ½ octave steps), we consider feature vectors **x** = [*x*_1_, *x*_2_, …, *x*_*N*_], consisting of principal component scores obtained from windowed shuffled correlations about 100 randomly selected time samples. We then evaluate the posterior probability of each neural response under each of the different distributions (one for each sound or category). The most probable case is chosen according to the maximum a posteriori (MAP) decision rule:

$$\text{S}=argmax_{m}\;P\left(m|x\right),$$

where S is the selected sound or category that maximizes the posteriori and *m* varies across all possible sounds or categories (*m* = 1 … 5 for paradigms 1 and 2, *m* = 1 … 3 for paradigm 3). In practice, we find the MAP sound or category by using the Bayes rule and maximizing the log-posterior, *L*. We assume that the sounds or categories are equiprobable a priori and that the features at each time sample are conditionally independent so that

$$L=log\left(P\left(m|x\right)\right)=log\left\lceil P\left(m\right)\overset{N}{\underset{n=1}{\prod }}\frac{P\left(x_{n}|m\right)}{P\left(x_{n}\right)}\right\rceil \propto \overset{N}{\underset{n=1}{\sum }}\log\left(P\left(x_{n}|m\right)\right)$$

To evaluate the contribution of temporal and spectral correlations for neural classification performance, we implement the neural classifier either using purely temporal, purely spectral, or joint spectro-temporal correlations. The purely spectral classifier only considers correlations at zero lag, ***c***_*spec*_(*t*) = *c*_*kl*_(*t*, 0), as the primary features (no time lag between different frequency channels). Note that *c*_*kl*_(*t*, 0) contains strictly frequency-dependent information because the recorded neural channels are tonotopically ordered and delays are removed. For the temporal classifier, we consider the correlations along the diagonal (*k* = *l*), ***c***_*temp*_(*t*) = *c*_*kk*_(*t*, *τ*), for delays extending between *τ* = −100 to 100 ms as the primary features. Next, we combined the spectral and temporal correlations and implemented a joint spectro-temporal classifier by model averaging:

$$L=log\left(P_{Spec-temp}\left(m|x\right)\right)=\alpha \cdot log\left(P_{spec}\left(m|x\right)\right)+\left(1-\alpha \right)\cdot log\left(P_{temp}\left(m|x\right)\right),$$

where 0 ≤ *a* ≤ 1 is a mixing coefficient that allows us to adjust the relative contribution of spectral and temporal correlations to the classifier. The value of alpha was optimized so as to maximize the log-likelihood for the spectro-temporal classifier.

We also implement a purely temporal correlation classifier that lacks frequency organization. Note that for the temporal correlation classifier described above, the features consist of the envelope autocorrelations taken across all possible frequency channels (***c***_*kk*_(*t*, *τ*)) and can convey tonotopic information through the identity of the recording channel *k*. In order to isolate purely temporal correlation cues, we remove the tonotopic information from the temporal correlation signal by randomly reordering the frequency channels of the validation data while maintaining the ordering in the model data during the classification step. This channel randomization assures that frequency-specific information from different channels is not available during the validation and only temporal information is used for the classification.

Finally, the classifier was applied separately for each of the acoustic paradigms, which allows us to test how neural correlations potentially contribute to distinct recognition tasks. In the case of paradigm 1, the correlation model was obtained directly from the model generation data half to the same sounds. For paradigm 2, the goal was to determine whether correlations on their own, irrespective of spectral cues, potentially contribute to neural representation. Furthermore, we were interested in determining to what extent the neural correlation structure is invariant to manipulations in the sound spectrum. For this reason, the model was generated using the original sounds, while the validation data were obtained from the responses to the 1/f equalized sound. Finally, for paradigm 3, the goal was to determine whether the neural correlations could distinguish sounds selected from multiple categories. For this reason, we fit the models using each category (as opposed to each individual sound) and assume that multiple exemplars and response trials are from the same likelihood distributions.

### Single-trial neural classifier

The noiseless neural classifiers described above (spectral, temporal, and spectro-temporal) were also implemented using single trials of the data. Whereas the noiseless classifiers set an upper bound on the classifier performance for the specific classification strategy (assuming noise is not present), the single-trial classifier contains noisy/variable neural data and instead provides a direct estimate of the classification performance from single observations. Thus, the single-trial classification is more akin to the strategies that might be employed while making real-world behavioral decisions.

The general classifier procedure for single trials was identical to the noiseless classifier, with the exception of the correlation feature vectors used for the model generation and the validation data segments, which now consist of unshuffled correlations for individual trials. During the random iterative selection of time segments of the model generation (using 1,000-ms windows) and validation data (varied window size), we concurrently randomized the selected trials, i.e., in addition to randomly selecting response segments at different time instances of the response, we simultaneously randomized the selected trials. Thus, whereas the feature vectors used for the noiseless classifier are based on noise-free estimates of the stimulus-driven correlations obtained using shuffled-correlogram procedures as defined above, the single-trial correlograms used for this classifier contain both signal and noise in which the noise correlations often tend to dominate in power (as shown in Results). In this case, the equivalent model spectro-temporal correlation, $c_{kl}^{total}\left(t,\tau \right)$, used to derive all feature vectors (spectral, temporal, or spectro-temporal) for the single-trial classifier is

$${\text{c}}_{kl}^{total}\left(t,\tau \right)={\text{c}}_{kl}^{stim}\left(t,\tau \right)+{\text{c}}_{kl}^{noise}\left(t,\tau \right)$$

(proof in S1 Text).

### Sound database for auditory model and classifier

Sounds representing 13 acoustic categories are obtained from a variety of digital sound sources. 195 sounds from 13 different acoustic categories are used to build distributions of dynamic, spectro-temporal correlation statistics of natural/man-made sounds. Sound segments are chosen so that they have minimal background noise and are drawn from three broad classes: vocalizations, environmental sounds, and man-made noises. Vocalizations include 1) single bird songs (various species), 2) cat meowing (single or multiple cats), 3) dog barking (single or multiple dogs), and 4) human speech (male speaker). Environmental sounds include 5) bird chorus (various species), 6) speech babble (in various environments, e.g., bars, supermarkets, squares), 7) fire, 8) thunder and rain, 9) flowing water (rivers and streams), 10) wave (ocean/lake waves), and 11) wind. Finally, man-made noises consist of 12) bell (church or tower bells) and 13) automobile engines (different vehicles). Each category contains 15 sounds, each 10 s long, sampled at *F*_*s*_ = 44.1 kHz (see S1 Table for sources and full list of tracks used).

### Auditory model

Sounds are analyzed through a cochlear filter bank model of the auditory periphery that decomposes the sound using frequency-organized cochlear filters. The cochlear filter bank consists of tonotopically arranged gamma-tone filters [58]. These filters have a sharp high-frequency cutoff and shallow low-frequency tails that resemble the tuning functions of auditory nerve fibers. The *k*-th gamma-tone filter has an impulse response function

$$h_{k}\left(t\right)=a_{k}t^{n-1}e^{-2\pi b_{k}t}\cos\left(2\pi f_{k}t+\phi \right),$$

where *k* is the filter channel, *t* denotes time, and *b*_*k*_ and *f*_*k*_ denote the filter bandwidth and center frequency. The filter gain coefficient *a*_*k*_ is chosen so that the filter passband gain is 1; filter order *n* and filter phase *ϕ* are 3 and 0, respectively. Filter bandwidths are chosen to follow perceptually derived critical bandwidths for humans, *b*_*k*_ = 25 + 75(1 +1.4*f*_*k*_^2^)^0.69^ [59,60]. We use *L* = 58 frequency channels with center frequencies *f*_*k*_ ranging from 100 Hz to 16 kHz in 1/8 octave steps. In the first stage of processing, the sound *s*(*t*) is passed through the cochlear filter bank model:

$$s_{k}\left(t\right)=s\left(t\right)*h_{k}\left(t\right),$$

where * represents the convolution operator. The outputs of the filter bank are next passed through a nonlinear envelope extraction stage, which models the characteristics of the hair cell. We first compute the magnitude of the analytic signal [61]:

$$s_{A,k}\left(t\right)=\left|s_{k}\left(t\right)+jH\left\{s_{k}\left(t\right)\right\}\right|,$$

where *H*{·} is the Hilbert transform operator and $j=\sqrt{-1}$. Then, the temporal envelope for each channel is obtained by convolving the rectified analytic signal magnitude with a b-spline low-pass filter with cutoff frequency of 500 Hz (transition bandwidth of 125 Hz and stopband attenuation of 60 dB):

$$S_{k}\left(t\right)=s_{A,k}\left(t\right)*h_{synapse}\left(t\right),$$

which models the low-pass filtering of the hair-cell synapse (*h*_*synapse*_(*t*)). The low-pass–filtered envelopes are then downsampled to 1 kHz for modeling. Here, we refer to the time-varying envelopes of the cochlear filters as the cochlear spectrogram and use the notation *S*(*t*, *f*_*k*_) = *S*_*k*_(*t*).

### Nonstationary spectro-temporal correlation statistics

Since natural sounds are often nonstationary, we measure not just the long-term correlation but the time-varying or “short-term” correlation statistics between the frequency-organized channels in the cochlear model representation. This nonstationary representation is then used to quantify the contribution of the sound-correlation statistics to sound categorization. The short-term correlation statistics that we use are similar to those for the neural data analysis except that we use the frequency channels from the cochlear spectrogram rather than the neural signals. The running short-term correlation function Φ between the cochlear spectrogram channel *k* and *l* is computed according to [32]

$$\Phi_{kl}\left(t,\tau \right)=\int_{-\infty }^{+\infty }S_{k}\left(\gamma \right)S_{l}\left(\gamma -\tau \right)W^{2}\left(t-\gamma \right)d\gamma ,$$

where *W*^2^(*γ*) is a sliding window function that determines the temporal resolution of the correlation measurement, *t* is the time, and *τ* is the cross-correlation delay. Here, *W*^2^(*γ*) is a Kaiser window (*β* = 3.4) where the overall window resolution (corresponding to two SDs of the Kaiser window width; varied between 25 to 566 ms in ½ octave steps) is varied to quantify the effects of the correlation temporal resolution on categorization performance. The range of permissible cross-correlation delays (−*τ*_*W*_ to +*τ*_*W*_) corresponds to half the window size and is thus varied between −12.5 to +12.5 for the highest resolution and −283 to +283 ms for the coarsest resolution. Conceptually, at each time point, the short-term correlation performs a correlation between the locally windowed envelope signals *S*_*k*_(*t*)*W*(*t* − *γ*) and *S*_*l*_(*t* − *τ*)*W*(*t* − *γ*) to estimate the localized correlation statistics of the cochlear envelopes.

To remove the influence of spectral power on the correlation measurements, the short-term correlations are normalized:

$${\text{c}}_{kl}\left(t,\tau \right)=\frac{\Phi_{kl}\left(t,\tau \right)}{\sqrt{\sigma_{k}^{2}\left(t\right)\cdot \sigma_{l}^{2}\left(t,\tau \right)}}$$

where

$$\sigma_{k}^{2}\left(t\right)=\int_{-\infty }^{+\infty }S_{k}\left(\gamma \right)W\left(t-\gamma \right)d\gamma$$

and

$$\sigma_{l}^{2}\left(t,\tau \right)=\int_{-\infty }^{+\infty }S_{l}\left(\gamma -\tau \right)W\left(t-\gamma \right)d\gamma$$

are the time-varying and delay-varying (for channel *l*) power *k*-th and *l*-th spectrogram channels. Again, as with a Pearson correlation coefficient, this short-term spectro-temporal correlation is bounded between −1 and 1. Unlike the neural correlation measures, *c*_*kl*_(*t*, 0) = 1 because there is no neural variability or noise in the model representation.

To assess the contribution of temporal and spectral correlations on the sound categorization performance, we perform a secondary analysis in which the spectro-temporal correlation function is decomposed into its purely spectral or temporal components, following a similar framework as for the neural data analysis. To evaluate the spectral correlations, we consider only the correlations at *τ* = 0 (no time lag between different frequency channels). For temporal correlations, *τ* ranges from 0 to up to half of the Kaiser window length. Only autocorrelation functions or correlation functions of a channel versus itself are considered in temporal correlation analysis, although all possible frequency channels are involved in spectral correlation analysis. Correlations between different frequency channels computed at zero time lag are referred to as purely spectral correlation components, whereas the autocorrelations computed at different time lags for each frequency channel are referred to as temporal correlation throughout.

### Stationarity and ensemble diversity indices

Given that sounds in our database are quite varied, ranging from isolated vocalizations to environmental sounds consisting of superposition of many individual acoustic events, we seek to characterize the overall degree of stationarity in the short-term correlation statistics for each of the ensemble. Furthermore, since sound recordings are obtained from different sources and animal species (e.g., for vocalizations), all of which could influence the overall category statistics, we also seek to quantify the overall diversity of the short-term correlation statistics of each ensemble. When considering sound categorization, we might expect that stationary sounds with minimal diversity across an ensemble would be most easily recognized.

For each sound, the sampled short-term spectro-temporal correlation *c*_*kl*_(*t*, *τ*) (computed using *τ*_*W*_ = 100 ms) is rearranged and expressed as a time-dependent vector function **$\overset{-}{c}\left(t\right)$** with dimensions *M* · *L*^2^ at each time point, where *M* = 99 is the number of time lags used for the short-term correlation and *L* is the number of frequency channels. The SI of each sound is defined and calculated as

$$SI=1-\frac{\langle \left\|\bar{c}\left(t\right)-\langle \bar{c}\left(t\right)\rangle \right\|\rangle }{\left\|\langle \bar{c}\left(t\right)\rangle \right\|}$$

where ||·|| is the vector norm and 〈·〉 is a time average. Conceptually, the second term in the SI equation corresponds to the time-averaged variance of the short-term correlation normalized by the total power of the time-averaged short-term correlation. As such, the SI measures the average normalized variability across time and is bounded between 0 and 1, where 1 indicates that the moment-to-moment variance of the short-term correlation is 0, thus indicating a high degree of stationarity. By comparison, when the moment-to-moment variance is high, the index approaches 0, indicating a highly nonstationary short-term correlation function.

We next define and measure the CDI, which is designed to measure the degree of homogeneity or heterogeneity in the short-term spectro-temporal correlation functions for each of the 13 sound categories studied. To do so, we first compute the time-averaged correlation function for each sound in a given ensemble, ${\overset{-}{c}}_{n}=\langle {\overset{-}{c}}_{n}\left(t\right)\rangle$, where *n* = 1…15 is an index representing the sounds for each sound category. The CDI is defined and computed as

$$CDI=\frac{\left\|{\bar{c}}_{n}-E\left[{\bar{c}}_{n}\right]\right\|}{\left\|E\left[{\bar{c}}_{n}\right]\right\|},$$

where *E*[·] is the expectation operator taken across the sound ensemble (equivalent to an average across sounds, *n*). Conceptually, the CDI corresponds to the variance of the time-averaged short-term correlation taken across the ensemble of sounds normalized by the power (norm) of the time and ensemble average short-term correlation.

For a particular sound category, a CDI near zero is indicative of low diversity (homogeneity) such that the short-term spectro-temporal correlation functions of that ensemble are quite similar from sound to sound and thus closely resemble the average ensemble correlations. By comparison, a CDI of 1 indicates a high degree of heterogeneity (high diversity) so that the short-term spectro-temporal correlations are quite different from sound to sound.

### Dimensionality reduction and distribution model

To reduce the dimensionality of the categorization problem, we use PCA. For spectral correlation statistics, the entries of the correlation matrix at zero time lag (*c*_*kl*_(*t*, 0)) are considered as features, while time points are used as observations or trials. For temporal correlation statistics, the correlations at different time lags within single-frequency channels (***c***_*kk*_(*t*, *τ*)) are considered features. Both time points and different frequency channels are treated as observations so that temporal information is not specific to any particular frequency channel. For further analysis, we use only the highest-ranked principal components that explain 90% of the variability in the data (26 principal components for spectral, 8 for temporal, 87 for spectro-temporal).

Using the low-dimensional representations of the spectral, temporal, or spectro-temporal correlations, we model the distributions of principal components for each sound category with a GMM. For each sound category *i*, we learn a multivariate probability distribution

$$P\left(x|m=i\right)=\overset{N_{c}}{\underset{k=1}{\sum }}a_{i,k}N\left(x;\mu_{i,k},C_{i,k}\right)$$

where *x* is the low-dimensional vector of PCA scores, *m* are the sound categories, *P*(***x***|*m*) is the multivariate PDF of sound features for category *m*, *a*_*k*_ ≥ 0 is the weight of the *k-*th mixture component, *N*(**x**; ***μ***_*i*,*k*_, **C**_*i*,*k*_) is the PDF of a multivariate normal distributions with mean ***μ***_*k*_ and covariance matrix **C**_*k*_, and *N*_*c*_ is the number of Gaussian components used for modeling. To avoid ill-conditioned covariance matrices, we constrain **C**_*k*_ to be diagonal.

In order to find the optimum number of Gaussian components (*N*_*c*_) required to model the data, we compute cross-validated likelihoods for different numbers of Gaussian mixtures (*N*_*c*_ = 1 to 20). The optimal *N*_*c*_ values are 5, 8, and 13 for temporal, spectral, and spectro-temporal, respectively.

### Bayesian classifier

Given the mixture model for each sound, we then use a Bayesian classifier for sound identification. The features fed to the classifier consist of the principal component scores from the sound’s short-term spectral, temporal, or spectro-temporal correlation. Since we are interested in how categorization performance change with the sound duration, we consider feature vectors ***x*** = [*x*_1_, *x*_2_, …, *x*_*N*_] consisting of principal component scores obtained from successive, windowed segments of sounds at different time samples (*t*_1 … *N*_) selected so that adjacent sound segments do not overlap. We then evaluate the posterior probability of each sound segment under the different mixture models. The most probable case is chosen according to the MAP decision rule:

$${\hat{m}}_{MAP}\left(x\right)=\underset{m}{argmax}P\left(m|x\right).$$

In practice, we find the MAP category by using the Bayes rule and maximizing the log-likelihood. We assume that the categories are equiprobable a priori and that the features at each time sample are conditionally independent. Thus, the MAP category is obtained by maximizing

$$log\left(P\left(m|x\right)\right)=log\left\lceil P\left(m\right)\prod_{n=1}^{N}\frac{P\left(x_{n}|m\right)}{P\left(x_{n}\right)}\right\rceil \propto \overset{N}{\underset{m=1}{?}}log\left(P\left(x_{n}|m\right)\right).$$

### Cross validation

To avoid overfitting, we use a leave-one-out cross validation in which all sounds are used to build the model distributions, except for one sound that is used for testing. Because there is only one sound validated, each validation iteration produces a 0% or 100% correct classification rate. The procedure is repeated iteratively over all sounds, and the average performance is obtained as the average classification rate across all iterations. The total sound duration used for the validation is varied by selecting *N* consecutive time window segments as described above from each sound under the test to be categorized; the selected *N*-segments start from the very beginning of the sound up to the end of the sound. Values of *N* are varied in ½ octave steps, starting with *N* = 1 up to the maximum value allowed by the sound duration.

### Optimal temporal resolution and integration time for categorizing sounds

As previously demonstrated for auditory neurons, an optimal temporal resolution can be identified for neural discrimination of natural sounds, and neural discrimination performance improves with the increasing sound duration [49,50]. For this reason, we seek to identify both the optimal temporal resolution that maximizes categorization performance as well as the integration time of the sound classifier. The temporal resolution of the correlation signals is varied by changing the sliding window temporal resolution, *τ*_*W*_, between 25–566 ms in ½ octave steps. Classification performance curves vary with *τ*_*W*_, exhibiting concave behavior with a clear maximum that is used to identify the optimal window time constant. The classifier performance also increased in an approximately exponential fashion with the overall sound duration. The classifier performance also increases with the overall sound duration. The classifier integration rise time, *τ*_*c*_, is defined as the amount of time required to achieve 90% of the asymptotic performance measured at 10 s duration.

### Spectrum-based classifier

In addition to estimating the contribution of the correlations structure towards categorization performance, we also used a spectrum classifier to estimate the contribution of the model spectrum for the categorization task. This condition serves as a reference control in part because the spectrum is normalized out of the correlation metrics, and it is possible that the correlation structure provides independent information towards the task. The spectrum-based classification procedure uses identical model generation and validation procedure as described above, with the exception of the model features used for the Bayesian classifier. Instead of using the spectral or temporal correlations as feature vectors, ***x***, we instead replaced correlation structure with the model spectrum by computing average amplitude of each output channel of the cochlear spectrogram. During the validation step, the spectrum was estimated iteratively for different window segments using identical windowing, temporal resolutions, *τ*_*W*_, and sound durations (by varying the number of consecutive time segments, *N*) as for the correlation classifier.

## Supporting information

S1 Text Main supporting information document containing mathematical proofs. (DOCX)

S1 Fig Correspondence between the model cochleogram and neural activity across 16 recording channels (neurogram). Cochleograms and neurograms are shown for the recording site shown in Fig 1 (five sounds used in paradigm 1). The fire cochleogram contains brief transient epochs with correlated transient events occurring at random times (ambers popping). Similarly, the neurogram shows sparsely activated and brief neural events with activation at comparable time instants. The neurogram for the bird chorus has periods of low neural activity that roughly correspond to periods with little sound power in the cochleogram (e.g., first approximately 250 ms). The crowd and water sounds have somewhat denser cochleograms and a corresponding dense response for channels 4–11. The cochleogram of the snake sound, by comparison, has periodic fluctuations around 8 kHz that produce a similar periodic activation for the high-frequency neural channels.
(DOCX)

S2 Fig Stimulus-driven and noise correlations for an example IC penetration site. Stimulus-driven spectral (A) and temporal (C) correlations for each site and the corresponding noise correlations (B, spectral; D, temporal). In general, noise correlations are localized in both time and frequency and do not exhibit stimulus-dependent structure. Stimulus-driven correlations are substantially more diverse and varied with the stimulus. IC, inferior colliculus
(DOCX)

S3 Fig Stimulus-driven and noise correlations for an example IC penetration site. The figure format is identical to S2 Fig. IC, inferior colliculus
(DOCX)

S4 Fig Stimulus-driven and noise correlations for an example IC penetration site. The figure format is identical to S2 Fig. IC, inferior colliculus
(DOCX)

S5 Fig Comparing the correlation structure of natural sounds to neural response correlations in IC ensembles. (A) Cross-channel envelope correlations for fire, bird, crowd, water, and snake sounds. The sound correlations are obtained by cross-correlating the frequency-organized outputs of a cochlear model representation. (B) Sound spectral correlations at selected frequency bands that match the neural best frequencies (measured at 65 dB SPL) of each recording channel for a representative IC penetration site. The gray contours in A indicate the selected frequency range for this representative recording location. (C) Stimulus-driven neural correlations of the corresponding IC recording site. (D) Boxplot of Pearson correlation coefficients between the frequency matched sound and neural correlations for five sounds (*N* = 13 penetration sites; diagonal terms are not included in the Pearson correlation coefficient calculation). Red boxplots indicate the actual measured correlation coefficient values (median = 0.37, 0.37, 0.25, 0.36, and 0.25, respectively) for same-sound comparisons (e.g., fire neural correlation versus fire sound correlation). Blue boxplots correspond control correlation coefficient values (median = −0.16, −0.17, 0.06, −0.13, 0.09) obtained across different-sound comparisons (e.g., fire neural correlation versus water, crowd, water, bird, and snake sound correlation). (E) Histogram plot of the Pearson correlation coefficients shown in (D; red = actual data; blue = different sound control). The average Pearson correlation coefficient is greater than zero and significantly different from the across-sound correlation coefficient control (red = 0.3 ± 0.03, for blue −0.04 ± 0.02, *p* = 1 × 10^−14^, one-tailed *t* test). IC, inferior colliculus; SPL, sound pressure level.
(DOCX)

S6 Fig Neural ensemble classification performance curves for individual sounds. Average results are shown for all penetration sites in the IC using the (A) spectro-temporal, (B) spectral, and (C) temporal classifiers as well as the (D) temporal classifier with frequency cues removed (no tonotopy). Red curves correspond to the population average of the single-trial classifier while blue represents the noiseless classifier. Red and blue filled boundaries represent ±1 SD. The spectral classifier performance trends across sounds closely mirrored the spectro-temporal classifier. The classifier performance is somewhat lower and more varied across sounds for the temporal classifier. (D) The performance is substantially reduced when tonotopic cues are removed from the temporal classifier. The classification accuracy of the single-trial classifier at 1 s duration exceeded chance (*p* < 0.05, *t* test) for all conditions except for the snake sound (*, temporal and temporal w/o tonotopy conditions, *p* = 0.15 and *p* = 0.92, respectively; *t* test). IC, inferior colliculus; w/o, without.
(DOCX)

S7 Fig Power spectrum of sound stimuli used for paradigms 2 and 3. (A) Original fire, bird, crowd, water, and snake sounds used for paradigm 2 have distinct power spectra (blue). (A) Spectrum-equalized variants for each sound have a 1/f power spectrum (red). These spectrum-equalized sounds can be readily identified even though the original and spectrum-equalized sounds can deviate by as much as 60 dB (e.g., crowd and water sounds). (B) The power spectrum of each of the six exemplars used for the fire, water, and speech categories (paradigm 3).
(DOCX)

S8 Fig Principal components obtained for the spectral and temporal correlation statistics of natural sounds. Principal components are shown in rank order according to the amount of variance accounted for. (A) First eight spectral principal components. Note that the diagonal has been removed since it is not informative (always takes on a value of 1). (B) First eight temporal principal components. The zero-lag component is not informative and has been removed (value of 1).
(DOCX)

S9 Fig The mixing coefficient of optimal spectro-temporal classifier for all IC penetration sites. The mixing coefficient (alpha) accounts for the relative weighting of spectral versus temporal cues used for the spectro-temporal classifier (see Materials and Methods). Values near 1 indicate that the classifier used strictly spectral cues for the identification or categorization task, while values near 0 indicate that the classifier uses purely temporal cues. For paradigms 1 (original) and 2 (spectrum-equalized), the median value of alpha is 0.6 (25th and 75th percentiles are 0.5 and 0.7), and for paradigm 3 (categorization), the median value of alpha is 0.5 (25th percentile is 0.4). This indicates that for both identification (paradigms 1 and 2) and categorization (paradigm 3), the optimal spectro-temporal classifier uses a mixture of spectral and temporal cues with roughly equal weighting. IC, inferior colliculus
(DOCX)

S10 Fig Comparing neural correlations obtained using aMUA and tMUA. tMUA spike trains (sequences of 0 s and 1 s) were first obtained by detecting signal events that exceeded 2 SDs above the recording noise floor. tMUA spike trains for different channels were then correlated to generate spectral and temporal correlation matrices (as for the aMUA). For a representative IC site, both spectral (A and B) and temporal (C and D) correlation patterns are very similar between aMUA and tMUA. However, the tMUA correlations (B and D) are weaker (see color scale) and noisier because they consist of sparse spike trains with random variability. (E) shows the Pearson correlation coefficients between aMUA- and tMUA-derived spectral and temporal correlations (Pearson correlation measured between the first and second half of the data). aMUA and tMUA spectral correlations are quite similar, as indicated by the relatively high Pearson correlation coefficient (tMUA versus aMUA, median = 0.81). For temporal correlations, the Pearson correlation between tMUA and aMUA was lower (median = 0.35), largely because of the noisy structure in the tMUA. (F) The reliability of the tMUA and aMUA activity was assessed by comparing neural correlations across halves of the data. The Pearson correlation coefficient comparing the first and second half of the aMUA activity (blue) is significantly higher for spectral (0.98) and temporal (0.83) correlations when compared against the tMUA (red; spectral = 0.77; temporal = 0.27). This indicates that aMUA is less noisy and thus more reliable across repeated trials of the stimulus. aMUA, analog multiunit activity; IC, inferior colliculus; tMUA, thresholded MUA
(DOCX)

S1 Movie Movie showing the short-term correlations for a barking dog. All movies follow the same format. The moving sliding window pans along the cochleogram from left to right with increasing time. All sounds are depicted for 100-ms window resolution (2 SD width of the analysis window; full window extent shown). The instantaneous spectral (left panel) and temporal (right panel) correlation for the windowed sound segment are shown below the cochleogram.
(MP4)

S2 Movie Movie showing the short-term correlations for running water. (MP4)

S3 Movie Movie showing the short-term correlations for speech. (MP4)

S4 Movie Movie showing the short-term correlations for white noise. (MP4)

S1 Sound Original sound sample used for neural recordings for paradigms 1 and 2. Sound = fire.
(WAV)

S2 Sound Original sound sample used for neural recordings for paradigms 1 and 2. Sound = bird.
(WAV)

S3 Sound Original sound sample used for neural recordings for paradigms 1 and 2. Sound = crowd.
(WAV)

S4 Sound Original sound sample used for neural recordings for paradigms 1 and 2. Sound = water.
(WAV)

S5 Sound Original sound sample used for neural recordings for paradigms 1 and 2. Sound = snake.
(WAV)

S6 Sound Spectrum-equalized sound variant used for neural recordings for paradigm 2. Derived directly from S1 Sound by synthetically changing the spectrum to 1/f spectrum. Sound = fire.
(WAV)

S7 Sound Spectrum-equalized sound variant used for neural recordings for paradigm 2. Derived directly from S2 Sound by synthetically changing the spectrum to 1/f spectrum. Sound = bird.
(WAV)

S8 Sound Spectrum-equalized sound variant used for neural recordings for paradigm 2. Derived directly from S3 Sound by synthetically changing the spectrum to 1/f spectrum. Sound = crowd.
(WAV)

S9 Sound Spectrum-equalized sound variant used for neural recordings for paradigm 2. Derived directly from S4 Sound by synthetically changing the spectrum to 1/f spectrum. Sound = water.
(WAV)

S10 Sound Spectrum-equalized sound variant used for neural recordings for paradigm 2. Derived directly from S5 Sound by synthetically changing the spectrum to 1/f spectrum. Sound = snake.
(WAV)

S1 Table List of sounds and sources used for the categorization model. (DOCX)

S2 Table List of sounds and sources used for neural categorization (sound paradigm 3). (DOCX)

## Abbreviations

aMUA: analog multiunit activity
CDI: category diversity index
GMM: Gaussian mixture model
IC: inferior colliculus
MAP: maximum a posteriori
PCA: principal component analysis
PSTH: poststimulus time histogram
SI: stationarity index
SNR: Signal-to-Noise Ratio
SPL: sound pressure level
