# Supplementary material for: A neural ensemble correlation code for sound category identification
Source: PLoS Biol. 2019 Oct 1;17(10):e3000449. doi: 10.1371/journal.pbio.3000449 (PMC6788721; doi:10.1371/journal.pbio.3000449)
Supplement: S1 Text — (DOCX) [file pbio.3000449.s001.docx]

**Supporting Information**

Proof: *Decomposing Signal and Noise Correlations*

In addition to measuring and examining the stimulus-driven correlations in the IC neural ensemble, we also measured the noise correlations. By definition, noise correlations correspond to correlated firing rate fluctuations across repeated presentations of a stimulus that are unrelated to the sensory signal (1, 2). Noise correlations are typically measured by correlating the firing rate residuals taken across repeated presentations of the stimulus (3). Within the IC, neurons can synchronize with precision of just a few milliseconds to the stimulus, and correlated activity is spatially organized (4) and can reflect the stimulus structure (5). For this reason, we developed an alternative framework using the shuffled and auto cross-correlograms between recording sites in order to measure the spatio-temporal noise correlations at the time-scales relevant to IC. For simplicity and purpose of the derivation, we assume that the shuffled and auto correlograms are obtained by averaging over infinite amount of time and trials, although the derivation extends to finite data as well as the short-term analysis performed in the manuscript.

We start by considering an additive noise model of the multi-site neural response across repeated presentations of the stimulus

| $r_{k,m}\left( t \right)=s_{k}\left( t \right)+n_{k,m}\left( t \right)$ |  |
| --- | --- |

where *k* is the recording site, *m* is the trial number, $s_{k}\left( t \right)$ is the stimulus-driven activity of the *k-th* recording site, and $n_{k,m}\left( t \right)$ is the recorded noise within the *k*-*th* recording site during the *m*-*th* response trial. It is assumed that noise reflects neural activity that is independent across trials and independent of the stimulus. As described in the main text, the shuffled cross-correlogram between the *k*-th and *l*-th recording site isolates the stimulus-driven correlations. It is obtained by taking the average cross-correlation between all possible response trials according to:

| $\Phi_{kl}^{shuffled}\left( \tau\right)=E\left[ \Phi_{mn,kl}\left( \tau\right) \right]=E\left[ \left\langle r_{k,m}\left( t \right)r_{l,n}\left( t-\tau\right) \right\rangle\right]$ |  |
| --- | --- |

where $\Phi_{mn,kl}\left( \tau\right)$ is the cross-correlation between recording channel (*k* and *l)* and the cross-correlations between recording sites are “shuffled” in the sense that they are taking across distinct trials *(*$n\neq m)$. The expectation, $E\left[ \cdot\right]$, is taken across all possible trial combinations while $\left\langle\cdot\right\rangle$ is the time average operator. Substituting the response model and expanding

| $\Phi_{kl}^{shuffled}\left( \tau\right)=E\left[ \left\langle s_{k}\left( t \right)s_{l}\left( t-\tau\right) \right\rangle\right]+E\left[ \left\langle s_{k}\left( t \right)n_{l,n}\left( t-\tau\right) \right\rangle\right]+E\left[ \left\langle n_{k,m}\left( t \right)s_{l}\left( t-\tau\right) \right\rangle\right]+E\left[ \left\langle n_{k,m}\left( t \right)n_{l,n}\left( t-\tau\right) \right\rangle\right]$ |  |
| --- | --- |

In the limiting case where the data length is sufficiently long, the independence assumption guarantees that the terms containing the noise approach zero and cancel out so that the shuffled correlogram strictly captures the stimulus-driven correlations:

| $\Phi_{kl}^{shuffled}\left( \tau\right)=\left\langle s_{k}\left( t \right)s_{l}\left( t-\tau\right) \right\rangle=\Phi_{kl}^{stim}\left( \tau\right)$ |  |
| --- | --- |

Next, we estimate the unshuffled cross-correlograms between recording sites

| $\Phi_{kl}^{unshuffled}\left( \tau\right)=E\left[ \Phi_{mm,kl}\left( \tau\right) \right]=E\left[ \left\langle r_{k,m}\left( t \right)r_{l,m}\left( t-\tau\right) \right\rangle\right].$ |  |
| --- | --- |

In the above, note that unlike the shuffled correlograms, the same trials are used to computed correlograms between recording sites. Upon substituting the response model we have

| $\Phi_{kl}^{unshuffled}\left( \tau\right)=E\left[ \left\langle\left( s_{k}\left( t \right)+n_{k,m}\left( t \right) \right)\left( s_{l}\left( t-\tau\right)+n_{l,m}\left( t-\tau\right) \right) \right\rangle\right]=E\left[ \left\langle s_{k}\left( t \right)s_{l}\left( t-\tau\right) \right\rangle\right]+E\left[ \left\langle s_{k}\left( t \right)n_{l,m}\left( t-\tau\right) \right\rangle\right]+E\left[ \left\langle n_{k,m}\left( t \right)s_{l}\left( t-\tau\right) \right\rangle\right]+E\left[ \left\langle n_{k,m}\left( t \right)n_{l,m}\left( t-\tau\right) \right\rangle\right]$ |  |
| --- | --- |

Note that in the limiting case, where the data length is sufficiently long, independence between the noise and signal assure that the correlation terms containing the noise and signal approach zero. Thus, we have

| $\boldsymbol{\Phi}_{\boldsymbol{kl}}^{\boldsymbol{unshuffled}}\left( \boldsymbol{\tau} \right)\boldsymbol{=E}\left[ \left\langle\boldsymbol{s}_{\boldsymbol{k}}\left( \boldsymbol{t} \right)\boldsymbol{s}_{\boldsymbol{l}}\left( \boldsymbol{t-\tau} \right) \right\rangle\right]\boldsymbol{+E}\left[ \left\langle\boldsymbol{n}_{\boldsymbol{k,m}}\left( \boldsymbol{t} \right)\boldsymbol{n}_{\boldsymbol{l,m}}\left( \boldsymbol{t-\tau} \right) \right\rangle\right]$  $\boldsymbol{=}\boldsymbol{\Phi}_{\boldsymbol{kl}}^{\boldsymbol{stim}}\left( \boldsymbol{\tau} \right)\boldsymbol{+}\boldsymbol{\Phi}_{\boldsymbol{kl}}^{\boldsymbol{noise}}\left( \boldsymbol{\tau} \right)$ |  |
| --- | --- |

Finally, we note that the noise correlations can be derived by subtracting the shuffled from the auto correlograms according to:

| $\boldsymbol{\Phi}_{\boldsymbol{kl}}^{\boldsymbol{noise}}\left( \boldsymbol{\tau} \right)\boldsymbol{=}\boldsymbol{\Phi}_{\boldsymbol{kl}}^{\boldsymbol{unshuffled}}\left( \boldsymbol{\tau} \right)\boldsymbol{-}\boldsymbol{\Phi}_{\boldsymbol{kl}}^{\boldsymbol{shuffled}}\left( \boldsymbol{\tau} \right)$ |  |
| --- | --- |

In order to compare noise and stimulus correlations using a common metric, the noise correlations are normalized by the total response power

| $\boldsymbol{c}_{\boldsymbol{kl}}^{\boldsymbol{noise}}\left( \boldsymbol{\tau} \right)\boldsymbol{=}\frac{\boldsymbol{\Phi}_{\boldsymbol{kl}}^{\boldsymbol{noise}}\left( \boldsymbol{\tau} \right)}{\sqrt{\boldsymbol{\sigma}_{\boldsymbol{k}}^{\boldsymbol{2}}\boldsymbol{\cdot}\boldsymbol{\sigma}_{\boldsymbol{l}}^{\boldsymbol{2}}}}\boldsymbol{=}\frac{\boldsymbol{\Phi}_{\boldsymbol{kl}}^{\boldsymbol{noise}}\left( \boldsymbol{\tau} \right)}{\sqrt{\left( \boldsymbol{\sigma}_{\boldsymbol{noise,k}}^{\boldsymbol{2}}\boldsymbol{+}\boldsymbol{\sigma}_{\boldsymbol{stim,k}}^{\boldsymbol{2}} \right)\boldsymbol{\cdot}\left( \boldsymbol{\sigma}_{\boldsymbol{noise,l}}^{\boldsymbol{2}}\boldsymbol{+}\boldsymbol{\sigma}_{\boldsymbol{stim,l}}^{\boldsymbol{2}} \right)}}$ |  |
| --- | --- |

where it is noted that the total variance contains both the signal and noise and noise variance, $\sigma_{k}^{2}=\sigma_{noise,k}^{2}+\sigma_{stim,k}^{2}$. In a similar fashion, the signal correlations are normalized by the response power

| $\boldsymbol{c}_{\boldsymbol{kl}}^{\boldsymbol{stim}}\left( \boldsymbol{\tau} \right)\boldsymbol{=}\frac{\boldsymbol{\Phi}_{\boldsymbol{kl}}^{\boldsymbol{stim}}\left( \boldsymbol{\tau} \right)}{\sqrt{\boldsymbol{\sigma}_{\boldsymbol{k}}^{\boldsymbol{2}}\boldsymbol{\cdot}\boldsymbol{\sigma}_{\boldsymbol{l}}^{\boldsymbol{2}}}}\boldsymbol{=}\frac{\boldsymbol{\Phi}_{\boldsymbol{kl}}^{\boldsymbol{stim}}\left( \boldsymbol{\tau} \right)}{\sqrt{\left( \boldsymbol{\sigma}_{\boldsymbol{noise,k}}^{\boldsymbol{2}}\boldsymbol{+}\boldsymbol{\sigma}_{\boldsymbol{stim,k}}^{\boldsymbol{2}} \right)\boldsymbol{\cdot}\left( \boldsymbol{\sigma}_{\boldsymbol{noise,l}}^{\boldsymbol{2}}\boldsymbol{+}\boldsymbol{\sigma}_{\boldsymbol{stim,l}}^{\boldsymbol{2}} \right)}}$ |  |
| --- | --- |

such that total normalized correlation

| $\boldsymbol{c}_{\boldsymbol{kl}}^{\boldsymbol{total}}\left( \boldsymbol{\tau} \right)\boldsymbol{=}\boldsymbol{c}_{\boldsymbol{kl}}^{\boldsymbol{stim}}\left( \boldsymbol{\tau} \right)\boldsymbol{+}\boldsymbol{c}_{\boldsymbol{kl}}^{\boldsymbol{noise}}\left( \boldsymbol{\tau} \right)$ |  |
| --- | --- |

is equivalent to correlation coefficient at zero lag and *k*=*l*, and is thus bounded between -1 to 1. That is, $c_{kl}^{total}\left( 0 \right)=1$, however, note that individually the stimulus and noise correlations at zero lag are both less than 1 ($c_{kl}^{stim}\left( 0 \right)<1$ and $c_{kl}^{noise}\left( 0 \right)<1$) because individually, they each only account for a fraction of the total response variance (which contains both signal and noise).

**Stimulus-driven and Noise Correlation Structure to Natural Sounds**

Stimulus-driven and noise correlations are shown for three additional penetration sites (S2-4 Figs.). Whereas the stimulus-driven correlations, both in time and frequency, are highly structured and can vary from stimulus to stimulus, the noise correlations for the three example penetration sites are substantially less structured. The spectral noise correlations (S2-S4 Figs., B) have strong diagonals indicating that noise correlations span all frequencies, although they are largely restricted to nearby frequency channels. The stimulus-driven spectral correlations (S2-S4 Figs., A) of all three penetration sites are substantially more varied across channels as well as across sounds. Stimulus-driven temporal correlations (S2-4 Figs., C) are also more varied and showed stimulus dependent structure when compared against the temporal noise correlations (S2-4 Figs., D). For instance, the bird vocalizations have a broad temporal component (particularly for S3 and S4 Figs.) while the rattling snake sound has a periodic component at ~20 Hz in all three penetration sites. The stimulus-driven temporal correlations for fire and water are substantially faster containing primarily a brief temporal component around zero lag for all frequencies. Finally, temporal noise correlations are overall highly restricted in time for all sounds lasting only a few milliseconds and show substantially less stimulus dependence than the corresponding stimulus-driven correlation.

*Neural Classifier Performance for Different Sounds*

The average neural population performance for each individual sound is shown in S4 Fig. Although similar average trends are observed for both the temporal and spectral neural correlation classifiers (Fig. 3), differences are observed between the performance for different sounds. For instance, the example of Fig. 3, the fire sound approaches ~100% classification accuracy for the temporal classifier regardless of temporal resolution used while the spectral classifier accuracy approaches ~80%. In general, we observe similar differences across the population where some sounds are better classified with temporal correlations compared to spectral correlations or vice versa (S4 Fig.). For example, fire classification accuracy is on average higher for the temporal classifier (Fig. 3C vs. B, ~80% temporal, ~50 % spectral). For the snake sound, by comparison, the trend is reversed (~50% accuracy for spectral, near chance for temporal). Examination of the neural correlations and the acoustic correlations in the rattling snake sound reveals that the periodicity of the snake rattle for the training (first half) and validation (second half) data has slightly different modulation frequencies (~16.5 Hz vs. 20 Hz) which leads to misclassification for a number of penetration sites. By comparison, the spectral correlation structure for this same sound is stable and sufficiently unique to allow high classification performance.

Audio Compilations

| [1] | *Atmospheres & Enviroments Sound Effects* [Sound Recording]. Sound Ideas Coorporation. <http://www.sound-ideas.com/>_­_. |
| --- | --- |
| [2] | *Sony Pictures Sound Effects Series Volumes 1-10. [Sound Recording]. Sony Corporation. 2003.* |
| [3] | *Sounds of Nature & The Great Outdoors. [Sound Recording]. Madacy Records. 1994.* |
| [4] | *Sounds of the Fascinating Animal World. [Sound Recording]. Madacy Records. 1994.* |
| [5] | *D. Stokes and L. Stokes, Composers, Stokes Field Guide to Bird Songs: Eastern Region. [Sound Recording]. Little, Brown & Company. 2010.* |
| [6] | *T. S. Schulenberg, Composer, Voices of Amazonian Birds, Vol. 1: Tinamous Through Barbets. [Sound Recording]. Cornell Laboratory Of Ornithology. 2000.* |
| [7] | *C. Davidson, Composer, Frog and Toad Calls of the Rocky Mountains: Vanishing Voices. [Sound Recording]. Cornell Laboratory Of Ornithology. 1996.* |

REFERENCES

1. Cohen MR, Newsome WT. Estimates of the contribution of single neurons to perception depend on timescale and noise correlation. J Neurosci. 2009;29(20):6635-48.

2. Abbott LF, Dayan P. The effect of correlated variability on the accuracy of a population code. Neural Comput. 1999;11(1):91-101.

3. Cohen MR, Kohn A. Measuring and interpreting neuronal correlations. Nat Neurosci. 2011;14(7):811-9.

4. Chen C, Read HL, Escabi MA. Precise feature based time-scales and frequency decorrelation lead to a sparse auditory code

. J Neurosci. 2012;32(25):8454-68.

5. Zheng Y, Escabi MA. Distinct roles for onset and sustained activity in the neuronal code for temporal periodicity and acoustic envelope shape. J Neurosci. 2008;28(52):14230-44.
