# Supplementary figures and images for: A neural ensemble correlation code for sound category identification
Source: PLoS Biol. 2019 Oct 1;17(10):e3000449. doi: 10.1371/journal.pbio.3000449 (PMC6788721; doi:10.1371/journal.pbio.3000449)

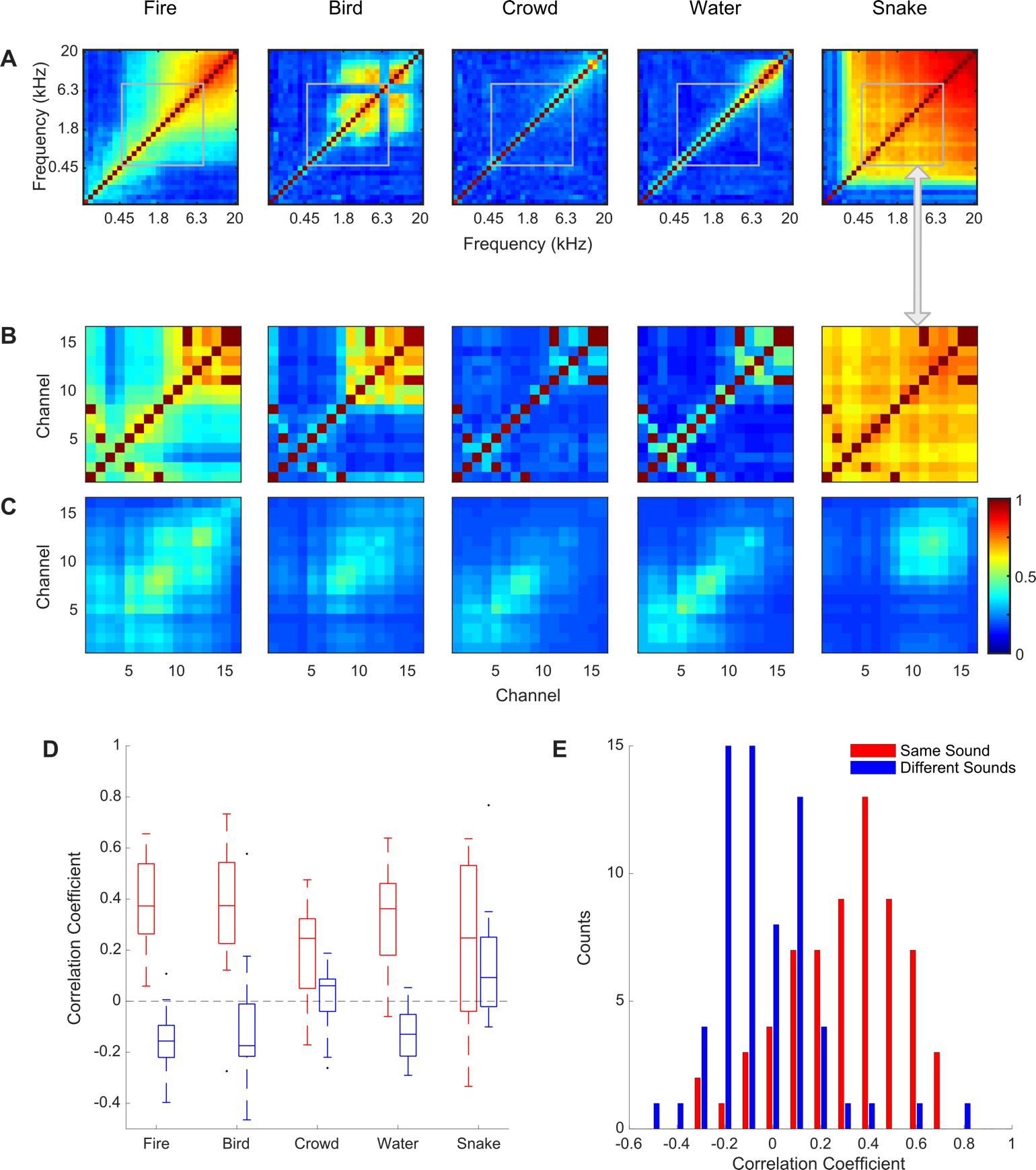

Supplement: S5 Fig — (A) Cross-channel envelope correlations for fire, bird, crowd, water, and snake sounds. The sound correlations are obtained by cross-correlating the frequency-organized outputs of a cochlear model representation. (B) Sound spectral correlations at selected frequency bands that match the neural best frequencies (measured at 65 dB SPL) of each recording channel for a representative IC penetration site. The gray contours in A indicate the selected frequency range for this representative recording location. (C) Stimulus-driven neural correlations of the corresponding IC recording site. (D) Boxplot of Pearson correlation coefficients between the frequency matched sound and neural correlations for five sounds (N = 13 penetration sites; diagonal terms are not included in the Pearson correlation coefficient calculation). Red boxplots indicate the actual measured correlation coefficient values (median = 0.37, 0.37, 0.25, 0.36, and 0.25, respectively) for same-sound comparisons (e.g., fire neural correlation versus fire sound correlation). Blue boxplots correspond control correlation coefficient values (median = −0.16, −0.17, 0.06, −0.13, 0.09) obtained across different-sound comparisons (e.g., fire neural correlation versus water, crowd, water, bird, and snake sound correlation). (E) Histogram plot of the Pearson correlation coefficients shown in (D; red = actual data; blue = different sound control). The average Pearson correlation coefficient is greater than zero and significantly different from the across-sound correlation coefficient control (red = 0.3 ± 0.03, for blue −0.04 ± 0.02, p = 1 × 10^−14^, one-tailed t test). IC, inferior colliculus; SPL, sound pressure level. (DOCX) [file pbio.3000449.s006.docx]

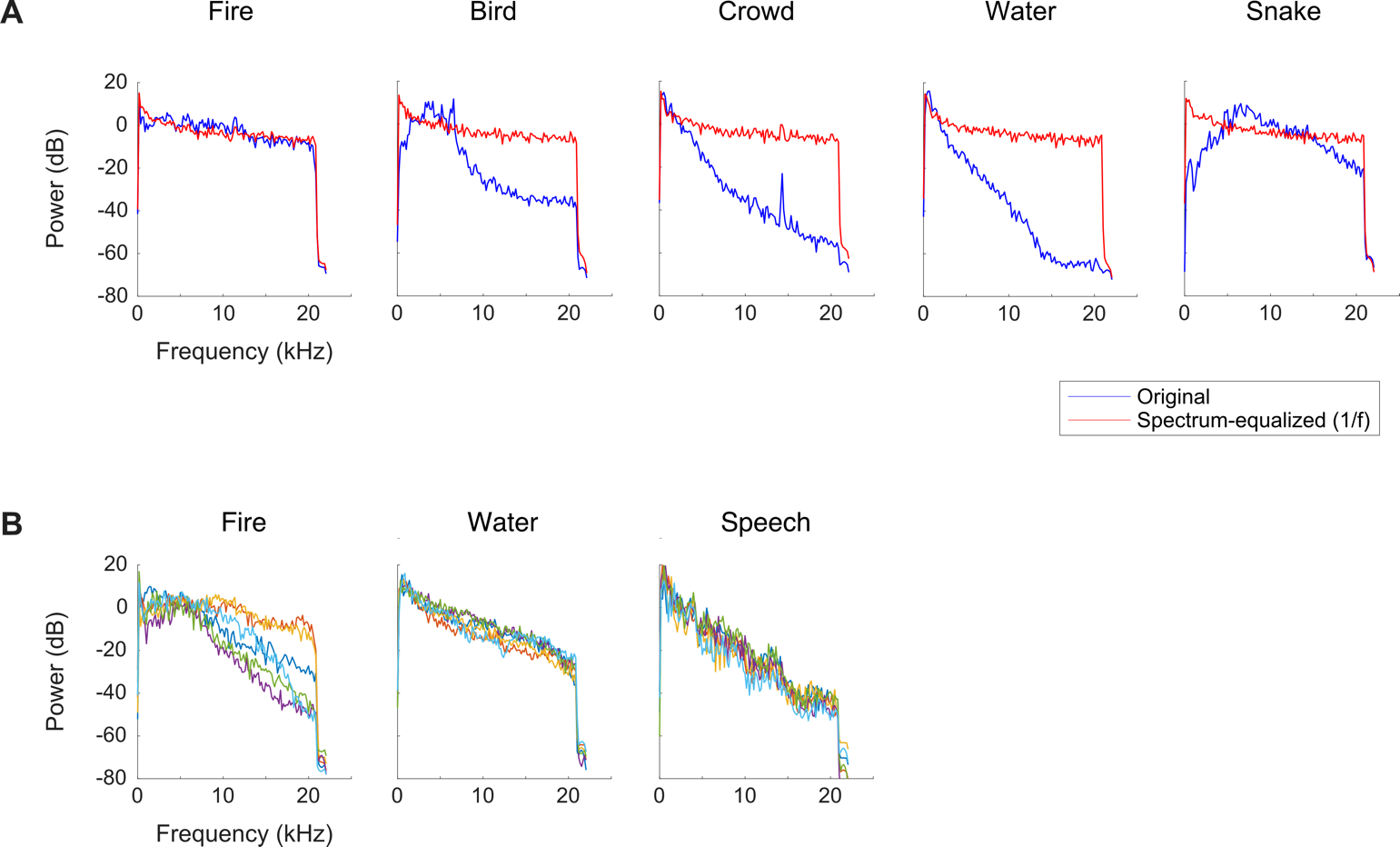

Supplement: S7 Fig — (A) Original fire, bird, crowd, water, and snake sounds used for paradigm 2 have distinct power spectra (blue). (A) Spectrum-equalized variants for each sound have a 1/f power spectrum (red). These spectrum-equalized sounds can be readily identified even though the original and spectrum-equalized sounds can deviate by as much as 60 dB (e.g., crowd and water sounds). (B) The power spectrum of each of the six exemplars used for the fire, water, and speech categories (paradigm 3). (DOCX) [file pbio.3000449.s008.docx]
