# Supplementary material for: A neural ensemble correlation code for sound category identification
Source: PLoS Biol. 2019 Oct 1;17(10):e3000449. doi: 10.1371/journal.pbio.3000449 (PMC6788721; doi:10.1371/journal.pbio.3000449)
Supplement: S1 Table — (DOCX) [file pbio.3000449.s026.docx]

**S1 Table.**

| Sound Category | Start Time (s) | Source Number | Volume/CD Number | Track Number/File Name |
| --- | --- | --- | --- | --- |
| Bell | 10 | [1] | - | Atmospheres and Environments 2496/BELL-CHURCH_GE­N-HDF-03189 |
| Bell | 11 | [1] | - | Atmospheres and Environments 2496/BELL-CHURCH_GEN-HDF-03198 |
| Bell | 3 | [1] | - | Atmospheres and Environments 2496/BELL-CHURCH_GEN-HDF-03191 |
| Bell | 10 | [1] | - | Atmospheres and Environments 2496/BELL-CHURCH_GEN-HDF-03195 |
| Bell | 3 | [1] | - | Atmospheres and Environments 2496/BELL-CLOCK-TOWER_GEN-HDF-03215 |
| Bell | 6 | [1] | - | Atmospheres and Environments 2496/BELL-CLOCK-TOWER_GEN-HDF-03220 |
| Bell | 32 | [1] | - | Atmospheres and Environments 2496/BELL-CLOCK-TOWER_GEN-HDF-03220 |
| Bell | 4 | [1] | - | Atmospheres and Environments 2496/BELL-CHURCH_GEN-HDF-03212 |
| Bell | 3 | [1] | - | Atmospheres and Environments 2496/BELL-CHURCH_GEN-HDF-03210 |
| Bell | 3 | [1] | - | Atmospheres and Environments 2496/BELL-CHURCH_GEN-HDF-03211 |
| Bell | 13 | [1] | - | Atmospheres and Environments 2496/BELL-CHURCH_GEN-HDF-03200 |
| Bell | 0 | [1] | - | Atmospheres and Environments 2496/BELL-CHURCH_GEN-HDF-03198 |
| Bell | 15 | [1] | - | Atmospheres and Environments 2496/BELL-CHURCH_GEN-HDF-03211 |
| Bell | 0 | [1] | - | Atmospheres and Environments 2496/BELL-CHURCH_GEN-HDF-03192 |
| Bell | 30 | [1] | - | Atmospheres and Environments 2496/BELL-CHURCH_GEN-HDF-03189 |
| Bird Chorus | 5 | [6] | 1 | ­096 |
| Bird Chorus | 5 | [6] | 1 | 095 |
| Bird Chorus | 1 | [6] | 1 | 094 |
| Bird Chorus | 4 | [6] | 1 | 046 |
| Bird Chorus | 2 | [6] | 1 | 022 |
| Bird Chorus | 5 | [1] | - | Atmospheres and Environments 2496/Residential-Day-Morning-Birds_GEN-HD2-31249 |
| Bird Chorus | 5 | [1] | - | Atmospheres and Environments 2496/Residential-Day-Morning-Birds_GEN-HD2-31248 |
| Bird Chorus | 5 | [1] | - | Atmospheres and Environments 2496/Residential-Day-Morning-Birds_GEN-HD2-31247 |
| Bird Chorus | 5 | [1] | - | Atmospheres and Environments 2496/Residential-Birds-Morning_GEN-HD2-31250 |
| Bird Chorus | 5 | [1] | - | Atmospheres and Environments 2496/Park-Ambience-Birds_GEN-HD2-31077 |
| Bird Chorus | 5 | [1] | - | Atmospheres and Environments 2496/Park-Ambience-Birds_GEN-HD2-31076 |
| Bird Chorus | 5 | [1] | - | Atmospheres and Environments 2496/Park-Ambience-Birds_GEN-HD2-31075 |
| Bird Chorus | 5 | [1] | - | Atmospheres and Environments 2496/FRANCE-BIRDS_GEN-HDF-12557 |
| Bird Chorus | 5 | [2] | 2 | Backgrounds/Birds Songs And Calls |
| Bird Chorus | 5 | [2] | 2 | Backgrounds/Forest Birds 01 |
| Single Bird Song | 2 | [5] | 3 | 007 |
| Single Bird Song | 2 | [5] | 3 | 013 |
| Single Bird Song | 2 | [5] | 3 | 016 |
| Single Bird Song | 2 | [5] | 3 | 017 |
| Single Bird Song | 2 | [5] | 3 | 021 |
| Single Bird Song | 2 | [5] | 3 | 037 |
| Single Bird Song | 9 | [5] | 3 | 073 |
| Single Bird Song | 8 | [5] | 3 | 092 |
| Single Bird Song | 13 | [5] | 2 | 055 |
| Single Bird Song | 2 | [5] | 2 | 038 |
| Single Bird Song | 2 | [5] | 2 | 085 |
| Single Bird Song | 2 | [5] | 2 | 095 |
| Single Bird Song | 2 | [5] | 3 | 098 |
| Single Bird Song | 9 | [5] | 1 | 078 |
| Single Bird Song | 2 | [5] | 1 | 073 |
| Cat Meowing | 0 | [4] | 1 | 002 |
| Cat Meowing | 10 | [4] | 1 | 003 |
| Cat Meowing | 0 | [4] | 1 | 005 |
| Cat Meowing | 0 | [4] | 1 | 006 |
| Cat Meowing | 0 | [4] | 1 | 018 |
| Cat Meowing | 4 | [4] | 1 | 019 |
| Cat Meowing | 0 | [2] | 1 | Animals/Cat Loud Meows |
| Cat Meowing | 13 | [4] | 1 | 002 |
| Cat Meowing | 5 | [1] | - | AnimalCat+6003_41_2 |
| Cat Meowing | 5 | [1] | - | Cat_DIGIC02-21 |
| Cat Meowing | 3 | [1] | - | Cat_DIGIC11-41 |
| Cat Meowing | 14 | [1] | - | Cat_DIGIC11-41 |
| Cat Meowing | 0 | [1] | - | Cat_DIGIVC1-39 |
| Cat Meowing | 3 | [1] | - | Cat+1002_03 |
| Cat Meowing | 0 | [1] | - | KittenMeowsSomeWav+PE530601 |
| Speech Babble | 2 | [2] | 8 | Backgrounds/Bar Interior 01 |
| Speech Babble | 3 | [1] | - | Atmospheres and Environments 2496/Spain-Crowd-Exterior-Square_GEN-HD2-31929 |
| Speech Babble | 3 | [1] | - | Atmospheres and Environments 2496/Spain-Crowd-Exterior-City-Centre_GEN-HD2-31927 |
| Speech Babble | 4 | [1] | - | Atmospheres and Environments 2496/Morocco-Crowd-Town-Square-Crowd_GEN-HD2-30911 |
| Speech Babble | 4 | [1] | - | Atmospheres and Environments 2496/Italy-Crowd-Cafe-Pedestrian-Area_GEN-HD2-30607 |
| Speech Babble | 10 | [1] | - | Atmospheres and Environments 2496/Germany-Crowd-Outdoor-Large_GEN-HD2-30132 |
| Speech Babble | 5 | [1] | - | Atmospheres and Environments 2496/England-Crowd-Outdoor-Pedestrians_GEN-HD2-29962 |
| Speech Babble | 5 | [1] | - | Atmospheres and Environments 2496/FINLAND-CROWD_GEN-HDF-11068 |
| Speech Babble | 8 | [1] | - | Atmospheres and Environments 2496/Africa-Crowd-Men-Loud_GEN-HD2-26319 |
| Speech Babble | 6 | [1] | - | Atmospheres and Environments 2496/Africa-Market-Outddor_GEN-HD2-26326 |
| Speech Babble | 7 | [1] | - | Atmospheres and Environments 2496/Germany-Crowd-Outdoor-Large_GEN-HD2-30127 |
| Speech Babble | 5 | [1] | - | Atmospheres and Environments 2496/ITALY-MARKET_GEN-HDF-16124 |
| Speech Babble | 7 | [1] | - | Atmospheres and Environments 2496/Netherlands-Bar-Outdoor_GEN-HD2-30986 |
| Speech Babble | 5 | [2] | 8 | Backgrounds/Cafe Interior |
| Speech Babble | 10 | [2] | 8 | Backgrounds/Bar Interior 02 |
| Dog Barking | 0 | [4] | 1 | 027 |
| Dog Barking | 18 | [4] | 1 | 027 |
| Dog Barking | 0 | [2] | 1 | Animals/Dog Large Barking |
| Dog Barking | 0 | [2] | 1 | Animals/Dog Medium Barking 01 |
| Dog Barking | 0 | [2] | 1 | Animals/Dog Small Barking |
| Dog Barking | 1 | [2] | 1 | Animals/Dogs Large Barking 01 |
| Dog Barking | 12 | [2] | 1 | Animals/Dogs Large Barking 01 |
| Dog Barking | 0 | [1] | - | AnimalDogCollie+6003_53_1 |
| Dog Barking | 0 | [1] | - | AnimalDogShepherd+6003_54_3 |
| Dog Barking | 0 | [1] | - | DogMedium+1002_19_2 |
| Dog Barking | 12 | [1] | - | DogMedium+1002_19_2 |
| Dog Barking | 3 | [1] | - | DogTerrier+2004_68 |
| Dog Barking | 0 | [1] | - | DogShepherd+2004_73_2 |
| Dog Barking | 1 | [1] | - | DogTerrier+2004_67_2 |
| Dog Barking | 12 | [1] | - | DogTerrier+2004_67_2 |
| Automobile Engine | 3 | [2] | 10 | Period Vehicles/1924 Ford Stake Bed Truck Ext Start Idle Away 01 |
| Automobile Engine | 3 | [2] | 10 | Period Vehicles/1924 Ford Stake Bed Truck Ext Start Idle Away 02 |
| Automobile Engine | 2 | [2] | 10 | Period Vehicles/1924 Ford Stake Bed Truck Ext Start Idle Fast Away |
| Automobile Engine | 3 | [2] | 10 | Period Vehicles/Ford Model T Away By Out |
| Automobile Engine | 3 | [2] | 10 | Period Vehicles/1954 Big Rig Int Start Steady Slow Idle Off |
| Automobile Engine | 5 | [2] | 10 | Period Vehicles/1954 Big Rig Int Start Rev Idle |
| Automobile Engine | 3 | [2] | 10 | Period Vehicles/1954 Big Rig Int Start Idle Revs Off |
| Automobile Engine | 3 | [2] | 10 | Period Vehicles/1954 Big Rig Int Start Idle Away Off |
| Automobile Engine | 3 | [2] | 10 | Period Vehicles/1954 Big Rig Ext Start Idle Reverse Off |
| Automobile Engine | 3 | [2] | 10 | Period Vehicles/1954 Big Rig Ext Start Idle Fast Away With Shifts |
| Automobile Engine | 10 | [2] | 10 | Period Vehicles/1954 Big Rig Ext Start Idle Away Slow Revs |
| Automobile Engine | 3 | [2] | 10 | Period Vehicles/1948 Diesel Truck Int Start Away Stop Idle Off |
| Automobile Engine | 3 | [2] | 10 | Period Vehicles/1948 Diesel Truck Ext Start Idle Reverse Idle Off |
| Automobile Engine | 4 | [2] | 10 | Period Vehicles/1937 Plymouth Ext Start Idle Off |
| Automobile Engine | 5 | [2] | 10 | Period Vehicles/1929 Sedan Int Start Idle Revs Off |
| Fire | 2 | [1] | - | Fire+6049_16 |
| Fire | 2 | [1] | - | FireCampfire+6015_11 |
| Fire | 2 | [1] | - | fireplace-wood-crackling_CAP01-184 |
| Fire | 2 | [2] | 8 | Backgrounds/Campfire 02 |
| Fire | 2 | [2] | 8 | Backgrounds/Fireplace Interior |
| Fire | 2 | [2] | 8 | Backgrounds/Campfire 01 |
| Fire | 2 | [2] | 7 | Impacts & Destruction Sweeteners/Constant Fire |
| Fire | 2 | [2] | 2 | Backgrounds/Fireworks Distant |
| Fire | 0 | [2] | 1 | Natural Elements/Fire Intense Crackle |
| Fire | 18 | [1] | - | Atmospheres and Environments 2496/Fire-Camp-Scene_GEN-HD2-30011 |
| Fire | 80 | [1] | - | Atmospheres and Environments 2496/Fire-Camp-Scene_GEN-HD2-30010 |
| Fire | 4 | [1] | - | Atmospheres and Environments 2496/FIRE-CAMP_GEN-HDF-11085 |
| Fire | 2 | [1] | - | fire-forest+burning_CAP01-183 |
| Fire | 2 | [1] | - | FIRE-CAMP_GEN-HDF-11090 |
| Fire | 2 | [1] | - | FireForest+6015_16 |
| Speech | 0 | [7] | 1 | 002 |
| Speech | 0 | [7] | 1 | 004 |
| Speech | 0 | [7] | 1 | 006 |
| Speech | 0 | [7] | 1 | 008 |
| Speech | 4 | [7] | 1 | 010 |
| Speech | 2 | [7] | 1 | 012 |
| Speech | 2 | [7] | 1 | 014 |
| Speech | 1 | [7] | 1 | 016 |
| Speech | 3 | [7] | 1 | 018 |
| Speech | 2 | [7] | 1 | 020 |
| Speech | 4 | [7] | 1 | 022 |
| Speech | 10 | [7] | 1 | 074 |
| Speech | 3 | [7] | 1 | 028 |
| Speech | 3 | [7] | 1 | 030 |
| Speech | 5 | [7] | 1 | 032 |
| Thunder Rain | 4 | [1] | - | Atmospheres and Environments 2496/THUNDER-RAIN_GEN-HDF-23325 |
| Thunder Rain | 0 | [1] | - | Atmospheres and Environments 2496/THUNDER-RAIN_GEN-HDF-23327 |
| Thunder Rain | 3 | [1] | - | Atmospheres and Environments 2496/THUNDER-RAIN_GEN-HDF-23328 |
| Thunder Rain | 38 | [1] | - | Atmospheres and Environments 2496/THUNDER-RAIN_GEN-HDF-23329 |
| Thunder Rain | 18 | [1] | - | Atmospheres and Environments 2496/THUNDER-RAIN_GEN-HDF-23334 |
| Thunder Rain | 0 | [1] | - | Atmospheres and Environments 2496/THUNDER-RAIN_GEN-HDF-23332 |
| Thunder Rain | 1 | [1] | - | Atmospheres and Environments 2496/THUNDER-RAIN_GEN-HDF-23333 |
| Thunder Rain | 3 | [1] | - | Atmospheres and Environments 2496/THUNDER-RAIN_GEN-HDF-23334 |
| Thunder Rain | 4 | [1] | - | Atmospheres and Environments 2496/THUNDER-RAIN_GEN-HDF-23342 |
| Thunder Rain | 22 | [1] | - | Atmospheres and Environments 2496/Thunder-Rain-Claps_GEN-HD2-32216 |
| Thunder Rain | 63 | [1] | - | Atmospheres and Environments 2496/THUNDER-RAIN_GEN-HDF-23335 |
| Thunder Rain | 18 | [1] | - | Atmospheres and Environments 2496/THUNDER-RAIN_GEN-HDF-23335 |
| Thunder Rain | 50 | [1] | - | Atmospheres and Environments 2496/THUNDER-RAIN_GEN-HDF-23336 |
| Thunder Rain | 25 | [1] | - | Atmospheres and Environments 2496/THUNDER-RAIN_GEN-HDF-23324 |
| Flowing Water | 3 | [1] | - | Atmospheres and Environments 2496/Water-Stream-Mountain_GEN-HD2-32607 |
| Flowing Water | 3 | [1] | - | Atmospheres and Environments 2496/Water-Stream-Culvert_GEN-HD2-32605 |
| Flowing Water | 3 | [1] | - | Atmospheres and Environments 2496/Water-Stream-Culvert_GEN-HD2-32604 |
| Flowing Water | 3 | [1] | - | Atmospheres and Environments 2496/Water-Stream-Culvert_GEN-HD2-32603 |
| Flowing Water | 3 | [1] | - | Atmospheres and Environments 2496/Water-Stream-Culvert_GEN-HD2-32602 |
| Flowing Water | 3 | [1] | - | Atmospheres and Environments 2496/Water-River-Mountain_GEN-HD2-32567 |
| Flowing Water | 3 | [1] | - | Atmospheres and Environments 2496/Water-River-Mountain_GEN-HD2-32566 |
| Flowing Water | 3 | [1] | - | Atmospheres and Environments 2496/WATER-STREAM_GEN-HDF-25654 |
| Flowing Water | 3 | [1] | - | Atmospheres and Environments 2496/WATER-STREAM_GEN-HDF-25653 |
| Flowing Water | 3 | [1] | - | Atmospheres and Environments 2496/WATER-STREAM_GEN-HDF-25652 |
| Flowing Water | 3 | [1] | - | Atmospheres and Environments 2496/Water-Stream-Over-Ice_GEN-HD2-32608 |
| Flowing Water | 3 | [3] | 1 | 003 |
| Flowing Water | 3 | [3] | 1 | 001 |
| Flowing Water | 3 | [1] | - | Atmospheres and Environments 2496/WATER-RIVER_GEN-HDF-25566 |
| Flowing Water | 3 | [1] | - | Atmospheres and Environments 2496/WATER-RIVER_GEN-HDF-25567 |
| Wave | 5 | [1] | - | Atmospheres and Environments 2496/WATER-OCEAN_GEN-HDF-25528 |
| Wave | 5 | [1] | - | Atmospheres and Environments 2496/WATER-OCEAN_GEN-HDF-25531 |
| Wave | 5 | [1] | - | Atmospheres and Environments 2496/WATER-OCEAN_GEN-HDF-25532 |
| Wave | 5 | [1] | - | Atmospheres and Environments 2496/Water-Ocean-Black-Sea_GEN-HD2-32552 |
| Wave | 5 | [1] | - | Atmospheres and Environments 2496/Water-Ocean-Black-Sea_GEN-HD2-32553 |
| Wave | 8 | [1] | - | Atmospheres and Environments 2496/Water-Ocean-Heavy-Break-Wall_GEN-HD2-32554 |
| Wave | 8 | [1] | - | Atmospheres and Environments 2496/Water-Ocean-Light-Break-Wall_GEN-HD2-32557 |
| Wave | 20 | [1] | - | Atmospheres and Environments 2496/Water-Ocean-Light-Break-Wall_GEN-HD2-32557 |
| Wave | 4 | [1] | - | Atmospheres and Environments 2496/WATER-LAKE_GEN-HDF-25521 |
| Wave | 5 | [1] | - | Atmospheres and Environments 2496/WATER-LAKE_GEN-HDF-25522 |
| Wave | 5 | [1] | - | Atmospheres and Environments 2496/WATER-LAKE_GEN-HDF-25524 |
| Wave | 5 | [1] | - | Atmospheres and Environments 2496/WATER-LAKE_GEN-HDF-25525 |
| Wave | 25 | [1] | - | Atmospheres and Environments 2496/WATER-LAKE_GEN-HDF-25525 |
| Wave | 30 | [1] | - | Atmospheres and Environments 2496/WATER-LAKE_GEN-HDF-25524 |
| Wave | 20 | [1] | - | Atmospheres and Environments 2496/Water-Ocean-Black-Sea_GEN-HD2-32552 |
| Wind | 3 | [1] | - | Atmospheres and Environments 2496/WIND-HOWL_GEN-HDF-25926 |
| Wind | 5 | [1] | - | Atmospheres and Environments 2496/WIND-HOWL_GEN-HDF-25927 |
| Wind | 5 | [1] | - | Atmospheres and Environments 2496/WIND-HOWL_GEN-HDF-25929 |
| Wind | 3 | [1] | - | Atmospheres and Environments 2496/WIND-HOWL_GEN-HDF-25928 |
| Wind | 5 | [1] | - | Atmospheres and Environments 2496/Wind-Whistling-Gusty_GEN-HD2-32724 |
| Wind | 3 | [1] | - | Atmospheres and Environments 2496/Wind-Whistle-Window_GEN-HD2-32727 |
| Wind | 5 | [1] | - | Atmospheres and Environments 2496/Wind-Whistle-Window_GEN-HD2-32726 |
| Wind | 5 | [1] | - | Atmospheres and Environments 2496/Wind-Whistle-Window_GEN-HD2-32725 |
| Wind | 5 | [1] | - | Atmospheres and Environments 2496/Wind-Mountain-Heavy_GEN-HD2-32718 |
| Wind | 5 | [1] | - | Atmospheres and Environments 2496/Wind-Mountain-Heavy_GEN-HD2-32717 |
| Wind | 5 | [2] | 1 | Natural Elements/Wind Strong |
| Wind | 5 | [2] | 1 | Natural Elements/Wind Medium |
| Wind | 5 | [2] | 8 | Wind/Violent Rustling Wind |
| Wind | 5 | [2] | 8 | Wind/Cold Wind |
| Wind | 60 | [2]­ | 1 | Natural Elements/Wind Medium |

Audio Compilations

| [1] | *Atmospheres & Enviroments Sound Effects* [Sound Recording]. Sound Ideas Coorporation. <http://www.sound-ideas.com/>_­_. |
| --- | --- |
| [2] | *Sony Pictures Sound Effects Series Volumes 1-10. [Sound Recording]. Sony Corporation. 2003.* |
| [3] | *Sounds of Nature & The Great Outdoors. [Sound Recording]. Madacy Records. 1994.* |
| [4] | *Sounds of the Fascinating Animal World. [Sound Recording]. Madacy Records. 1994.* |
| [5] | *D. Stokes and L. Stokes, Composers, Stokes Field Guide to Bird Songs: Eastern Region. [Sound Recording]. Little, Brown & Company. 2010.* |
| [6] | *T. S. Schulenberg, Composer, Voices of Amazonian Birds, Vol. 1: Tinamous Through Barbets. [Sound Recording]. Cornell Laboratory Of Ornithology. 2000.* |
| [7] | *C. Davidson, Composer, Frog and Toad Calls of the Rocky Mountains: Vanishing Voices. [Sound Recording]. Cornell Laboratory Of Ornithology. 1996.* |
