# Supplementary material for: A neural ensemble correlation code for sound category identification
Source: PLoS Biol. 2019 Oct 1;17(10):e3000449. doi: 10.1371/journal.pbio.3000449 (PMC6788721; doi:10.1371/journal.pbio.3000449)
Supplement: S2 Table — (DOCX) [file pbio.3000449.s027.docx]

**S2 Table.**

| Sound Category | Start Time (s) | Source Number | Volume/CD Number | Track Number/File Name |
| --- | --- | --- | --- | --- |
| Fire | 2 | [1] | - | Fire+6049_16 |
| Fire | 2 | [1] | - | FireCampfire+6015_11 |
| Fire | 2 | [1] | - | fireplace-wood-crackling_CAP01-184 |
| Fire | 2 | [2] | 8 | Backgrounds/Campfire 02 |
| Fire | 2 | [2] | 8 | Backgrounds/Campfire 01 |
| Fire | 0 | [2] | 1 | Natural Elements/Fire Intense Crackle |
| Water | 3 | [1] | - | Atmospheres and Environments 2496/Water-Stream-Mountain_GEN-HD2-32607 |
| Water | 3 | [1] | - | Atmospheres and Environments 2496/Water-River-Mountain_GEN-HD2-32567 |
| Water | 3 | [1] | - | Atmospheres and Environments 2496/WATER-STREAM_GEN-HDF-25654 |
| Water | 3 | [1] | - | Atmospheres and Environments 2496/WATER-STREAM_GEN-HDF-25653 |
| Water | 3 | [1] | - | Atmospheres and Environments 2496/WATER-STREAM_GEN-HDF-25652 |
| Water | 3 | [1] | - | Atmospheres and Environments 2496/Water-Stream-Culvert_GEN-HD2-32603 |
| Speech | 0 | [7] | 1 | 008 |
| Speech | 4 | [7] | 1 | 010 |
| Speech | 1 | [7] | 1 | 016 |
| Speech | 10 | [7] | 1 | 074 |
| Speech | 5 | [7] | 1 | 032 |
| Speech | 2 | [7] | 1 | 012 |

Audio Compilations

| [1] | *Atmospheres & Enviroments Sound Effects* [Sound Recording]. Sound Ideas Coorporation. <http://www.sound-ideas.com/>_­_. |
| --- | --- |
| [2] | *Sony Pictures Sound Effects Series Volumes 1-10. [Sound Recording]. Sony Corporation. 2003.* |
| [3] | *Sounds of Nature & The Great Outdoors. [Sound Recording]. Madacy Records. 1994.* |
| [4] | *Sounds of the Fascinating Animal World. [Sound Recording]. Madacy Records. 1994.* |
| [5] | *D. Stokes and L. Stokes, Composers, Stokes Field Guide to Bird Songs: Eastern Region. [Sound Recording]. Little, Brown & Company. 2010.* |
| [6] | *T. S. Schulenberg, Composer, Voices of Amazonian Birds, Vol. 1: Tinamous Through Barbets. [Sound Recording]. Cornell Laboratory Of Ornithology. 2000.* |
| [7] | *C. Davidson, Composer, Frog and Toad Calls of the Rocky Mountains: Vanishing Voices. [Sound Recording]. Cornell Laboratory Of Ornithology. 1996.* |
