# Supplementary material for: A neural ensemble correlation code for sound category identification
Source: PLoS Biol. 2019 Oct 1;17(10):e3000449. doi: 10.1371/journal.pbio.3000449 (PMC6788721; doi:10.1371/journal.pbio.3000449)
Supplement: Supplementary file 5 [file pbio.3000449.s002.docx]

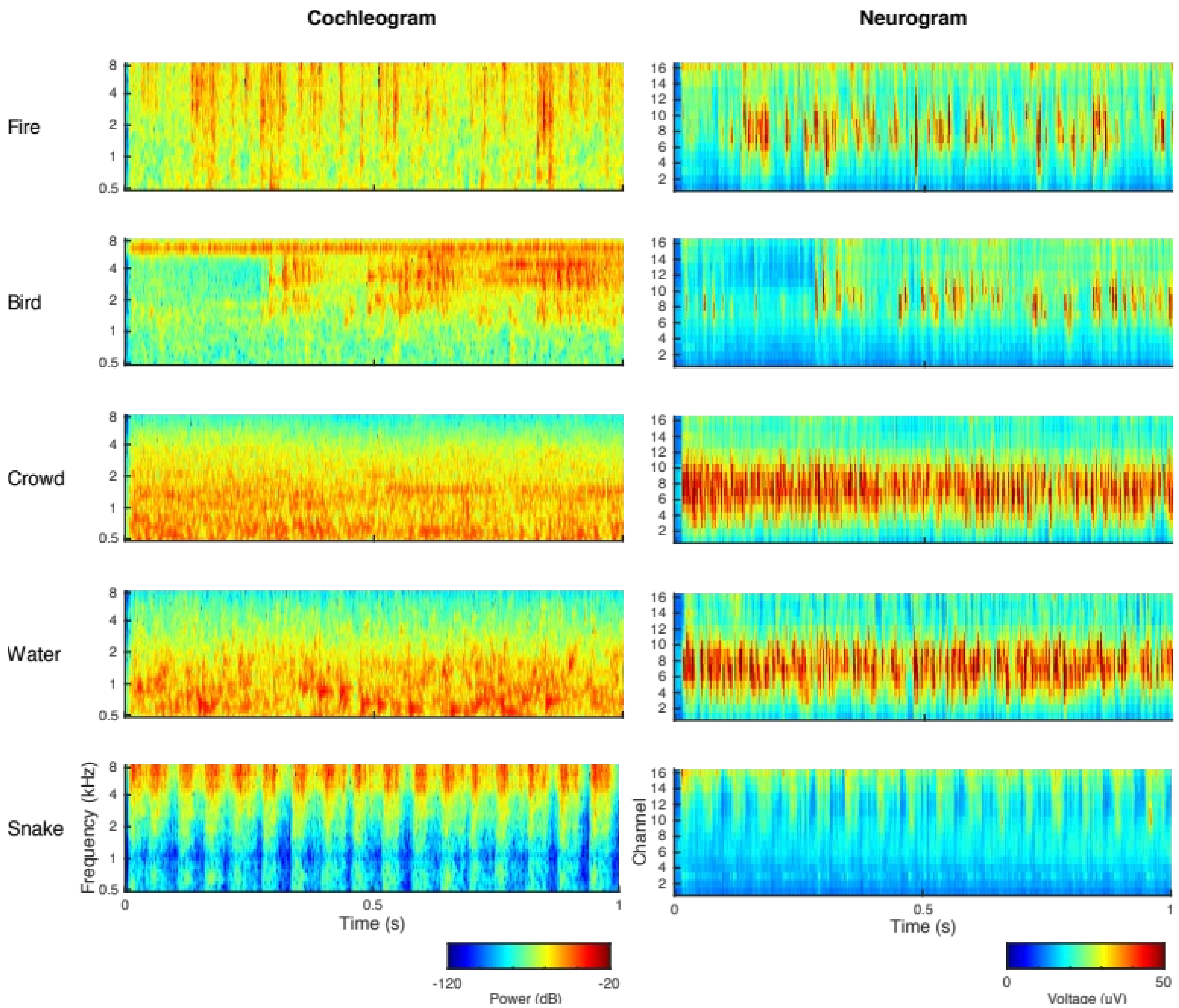

Cochleogram
Neurogram
Fire
Bird
Crowd
Water
Snake
Frequency (kHz)
Channel
Time (s)
Time (s)
-120
-20
Power (dB)
0
50
Voltage (uV)
