# Supplementary material for: A neural ensemble correlation code for sound category identification
Source: PLoS Biol. 2019 Oct 1;17(10):e3000449. doi: 10.1371/journal.pbio.3000449 (PMC6788721; doi:10.1371/journal.pbio.3000449)
Supplement: Supplementary file 6 [file pbio.3000449.s003.docx]

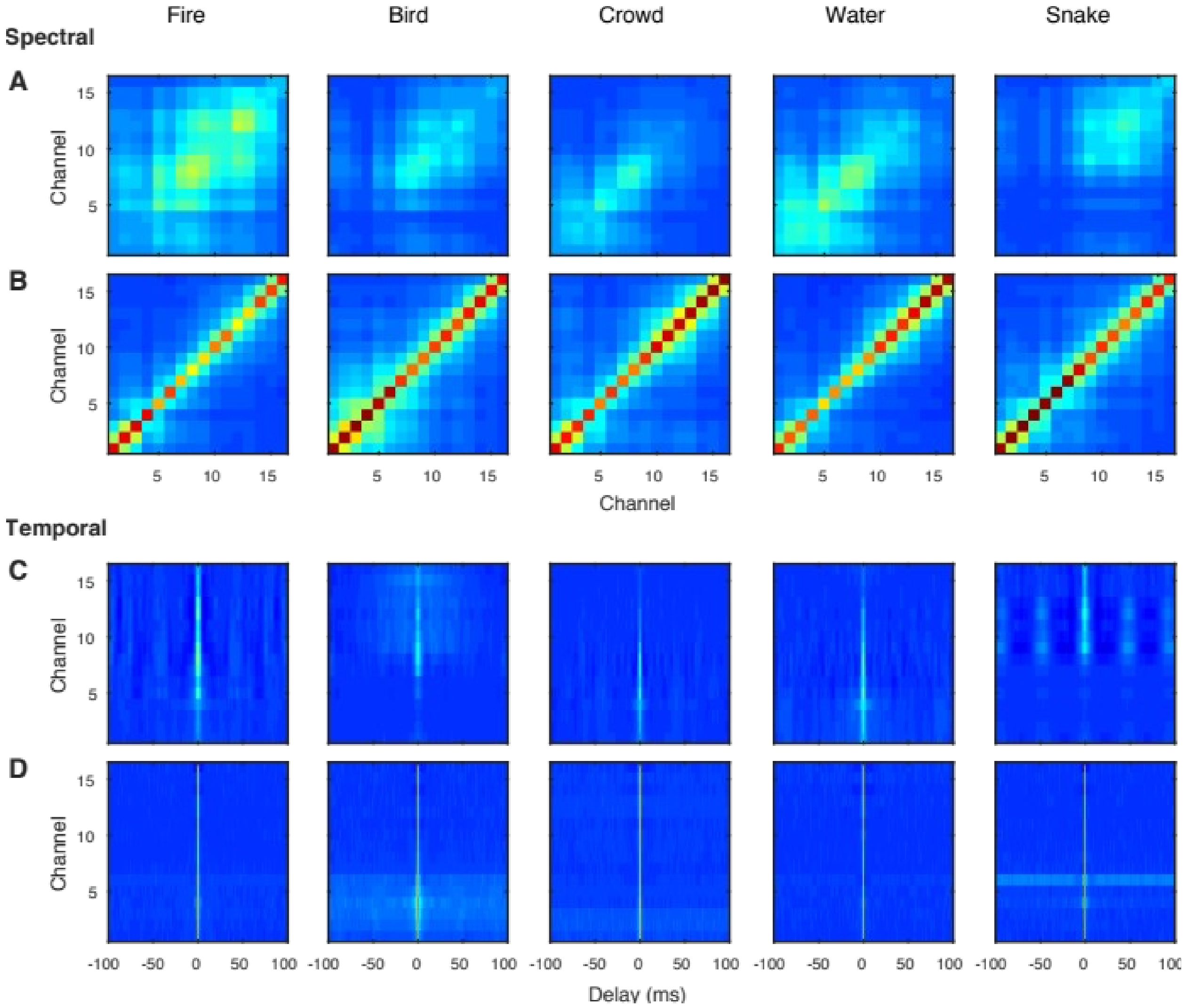

Fire
Bird
Crowd
Water
Snake
Spectral
A
B
Channel
5
10
15
Temporal
C
D
-100
-50
0
50
100
Delay (ms)
