# Supplementary material for: A neural ensemble correlation code for sound category identification
Source: PLoS Biol. 2019 Oct 1;17(10):e3000449. doi: 10.1371/journal.pbio.3000449 (PMC6788721; doi:10.1371/journal.pbio.3000449)
Supplement: Supplementary file 9 [file pbio.3000449.s007.docx]

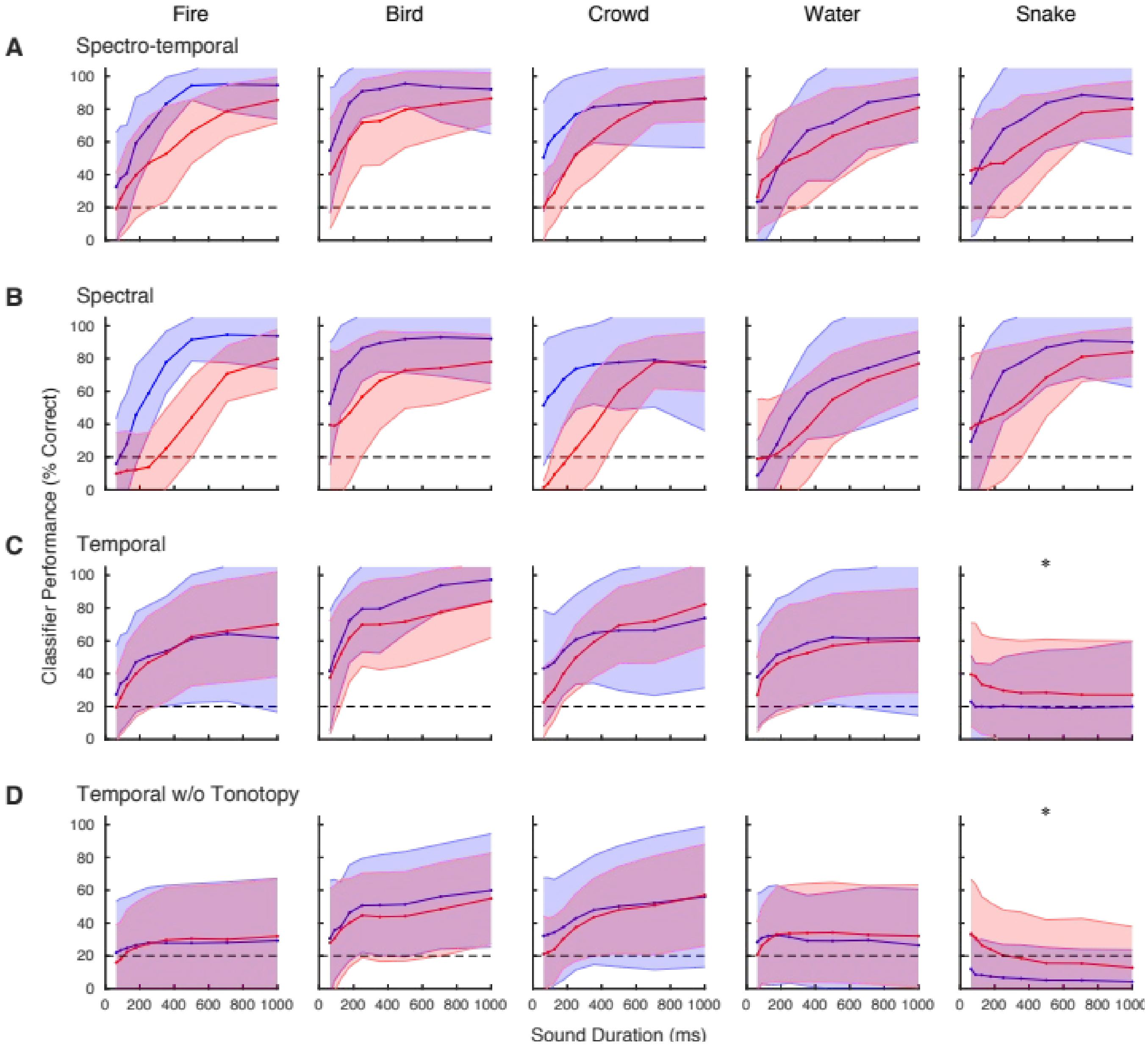

Fire
Bird
Crowd
Water
Snake
A
Spectro-temporal
B
Spectral
C
Temporal
D
Temporal w/o Tonotopy
Classifier Performance (% Correct)
Sound Duration (ms)
0
20
40
60
80
100
200
400
600
800
1000
*
