# Supplementary material for: A neural ensemble correlation code for sound category identification
Source: PLoS Biol. 2019 Oct 1;17(10):e3000449. doi: 10.1371/journal.pbio.3000449 (PMC6788721; doi:10.1371/journal.pbio.3000449)
Supplement: Supplementary file 10 [file pbio.3000449.s009.docx]

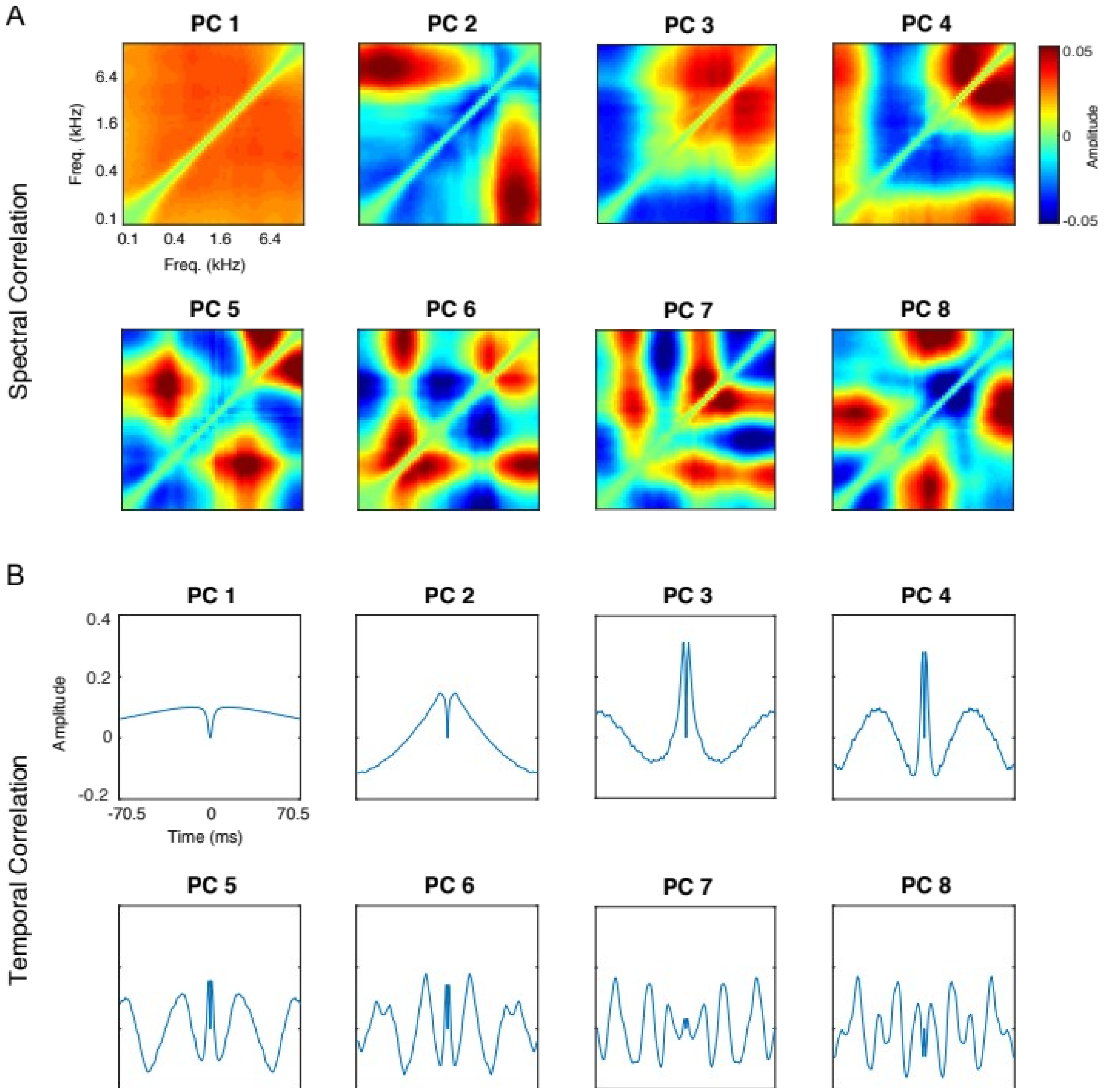
A
Spectral Correlation
PC 1
PC 2
PC 3
PC 4
6.4
1.6
0.4
0.1
Freq. (kHz)
0.1
0.4
1.6
6.4
Freq. (kHz)
0.05
0
-0.05
Amplitude
PC 5
PC 6
PC 7
PC 8
B
Temporal Correlation
PC 1
PC 2
PC 3
PC 4
0.4
0.2
0
-0.2
Amplitude
-70.5
0
70.5
Time (ms)
PC 5
PC 6
PC 7
PC 8
