# Supplementary material for: A neural ensemble correlation code for sound category identification
Source: PLoS Biol. 2019 Oct 1;17(10):e3000449. doi: 10.1371/journal.pbio.3000449 (PMC6788721; doi:10.1371/journal.pbio.3000449)
Supplement: Supplementary file 11 [file pbio.3000449.s010.docx]

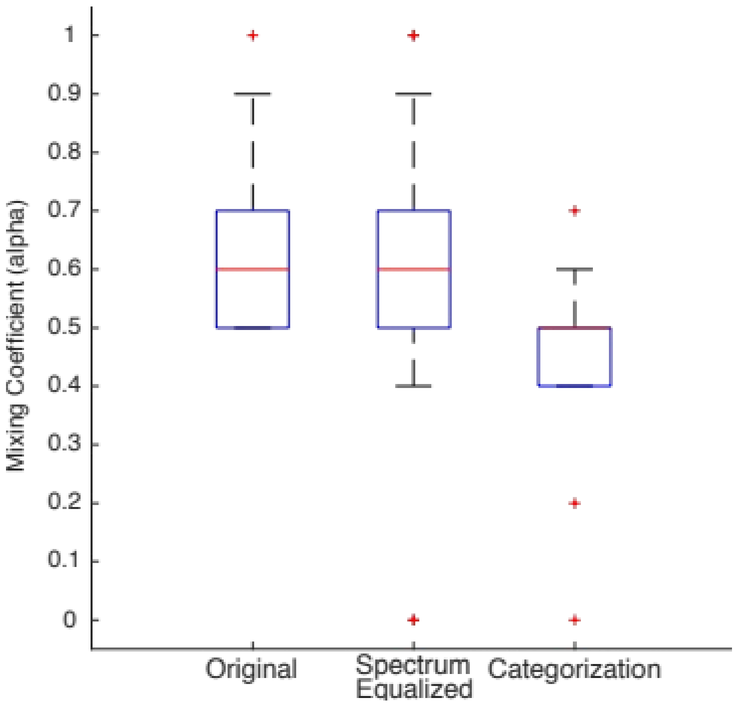

Mixing Coefficient (alpha)
1
0.9
0.8
0.7
0.6
0.5
0.4
0.3
0.2
0.1
0
Original
Spectrum Equalized
Categorization
