# Supplementary material for: A neural ensemble correlation code for sound category identification
Source: PLoS Biol. 2019 Oct 1;17(10):e3000449. doi: 10.1371/journal.pbio.3000449 (PMC6788721; doi:10.1371/journal.pbio.3000449)
Supplement: Supplementary file 12 [file pbio.3000449.s011.docx]

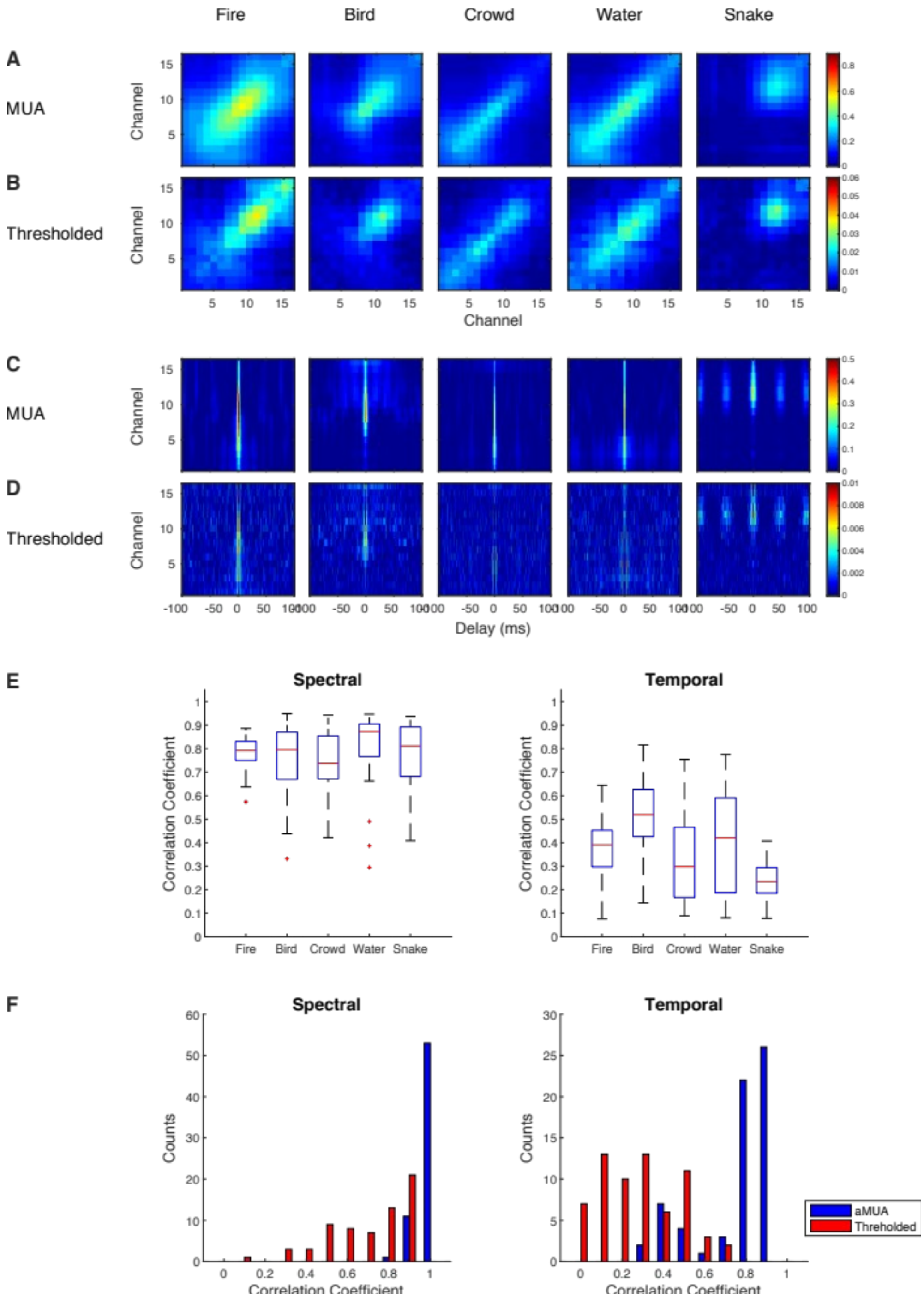
Fire
Bird
Crowd
Water
Snake
A
MUA
B
Thresholded
Channel
C
MUA
D
Thresholded
Delay (ms)
E
Spectral
Temporal
Correlation Coefficient
F
Spectral
Temporal
Counts
Correlation Coefficient
aMUA
Threholded
